# A data-driven framework for selecting and validating digital health metrics: use-case in neurological sensorimotor impairments

**DOI:** 10.1038/s41746-020-0286-7

**Published:** 2020-05-29

**Authors:** Christoph M. Kanzler, Mike D. Rinderknecht, Anne Schwarz, Ilse Lamers, Cynthia Gagnon, Jeremia P. O. Held, Peter Feys, Andreas R. Luft, Roger Gassert, Olivier Lambercy

**Affiliations:** 1 Rehabilitation Engineering Laboratory, Institute of Robotics and Intelligent Systems, Department of Health Sciences and Technology, ETH Zurich, Switzerland; 2 Division of Vascular Neurology and Rehabilitation, Department of Neurology, University Hospital and University of Zürich, Zurich, Switzerland; 3 Cereneo Center for Neurology and Rehabilitation, Vitznau, Switzerland; 4 REVAL, Rehabilitation Research Center, BIOMED, Biomedical Research Institute, Faculty of Medicine and Life Sciences, Hasselt University, Diepenbeek, Belgium; 5 Rehabilitation and MS Center, Pelt, Belgium; 6 School of Rehabilitation, Faculty of Medicine and Health Sciences, Université de Sherbrooke, Québec, Canada

**Keywords:** Diagnostic markers, Predictive markers, Prognostic markers, Multiple sclerosis, Neurological disorders

## Abstract

Digital health metrics promise to advance the understanding of impaired body functions, for example in neurological disorders. However, their clinical integration is challenged by an insufficient validation of the many existing and often abstract metrics. Here, we propose a data-driven framework to select and validate a clinically relevant core set of digital health metrics extracted from a technology-aided assessment. As an exemplary use-case, the framework is applied to the Virtual Peg Insertion Test (VPIT), a technology-aided assessment of upper limb sensorimotor impairments. The framework builds on a use-case-specific pathophysiological motivation of metrics, models demographic confounds, and evaluates the most important clinimetric properties (discriminant validity, structural validity, reliability, measurement error, learning effects). Applied to 77 metrics of the VPIT collected from 120 neurologically intact and 89 affected individuals, the framework allowed selecting 10 clinically relevant core metrics. These assessed the severity of multiple sensorimotor impairments in a valid, reliable, and informative manner. These metrics provided added clinical value by detecting impairments in neurological subjects that did not show any deficits according to conventional scales, and by covering sensorimotor impairments of the arm and hand with a single assessment. The proposed framework provides a transparent, step-by-step selection procedure based on clinically relevant evidence. This creates an interesting alternative to established selection algorithms that optimize mathematical loss functions and are not always intuitive to retrace. This could help addressing the insufficient clinical integration of digital health metrics. For the VPIT, it allowed establishing validated core metrics, paving the way for their integration into neurorehabilitation trials.

## Introduction

Assessments of impaired body functions, as observed in many diseases and disorders, are a fundamental part of the modern healthcare system^1^. Specifically, these assessments are essential to shed light on the often unknown mechanisms underlying the impairments and their temporal evolution, to individualize therapeutic interventions, and to provide documentation for insurances justifying further therapy. An exemplary application scenario of assessments are neurological disorders, including stroke, multiple sclerosis (MS), and hereditary ataxic conditions, where impairments in the sensorimotor system are commonly present, for example, when coordinating arm and hand during goal-directed activities^2–5^. In research studies, such deficits are often assessed by healthcare practitioners, who subjectively evaluate persons with impairments during multiple standardized tasks (referred to as conventional scales)^6–8^. While most of these scales are validated and their interpretation fairly well understood and documented, they often have a limited ability to detect fine impairments because of limited knowledge about behavioral variability, low resolution, and ceiling effects^9,10^. This can lead to bias when attempting to model and better understand longitudinal changes in impairment severity^11,12^.

Digital health metrics, herein defined as discrete one-dimensional metrics that are extracted from health-related sensor data, promise to overcome these shortcomings by proposing objective and traceable descriptions of human behavior without ceiling effects and with high resolution^13–17^. This offers the potential to more sensitively characterize impairments and significantly reduce sample sizes required in resource-demanding clinical trials^18^. In the context of assessing sensorimotor impairments, a variety of digital health metrics relying on kinematic or kinetic data have been successfully applied to characterize abnormal movement patterns^13,19,20^.

However, the integration of digital health metrics into clinical routine and research is still inhibited by an insufficient evaluation of the vast amount of existing measures and the need for core sets of validated and clinically relevant measures for the targeted impairments^13,21–23^. Indeed, recent reviews reported the use of over 150 sensor-based metrics for quantifying upper limb sensorimotor impairments and highlighted a clear lack of evidence regarding their pathophysiological motivation and clinimetric properties^13,24^. Especially the ability of a metric to detect impairments (discriminant validity) as well as the dependency to other metrics and the underlying information content (structural validity) are often not evaluated. Similarly, test–retest reliability, measurement error arising from intra-subject variability, and learning effects are only rarely considered, but their evaluation is fundamental to reliably and sensitively quantify impairments in an insightful manner^25^. Further, the influence of participant demographics, such as age, sex, and handedness, on the metrics is often not accurately modeled, but needs to be taken into account to remove possible confounds and provide an unbiased assessment. Most importantly, the high variability of clinimetric properties across behavioral tasks and sensor-based metrics motivates the need for a methodology to select metrics for a specific assessment task, starting from a large set of potential metrics that should be narrowed down to a clinically relevant core set^13,21,26^. Existing approaches to select core sets of metrics commonly rely on the consensus from a group of selected experts, which can lead to bias and is often not task-dependent^21,27–30^. Moreover, existing data-driven selection procedures (e.g., regression-based methods such as LASSO), are rarely tailored to the specific requirements of digital health metrics, where often no accurate ground truth about the targeted impairments is available^31–33^. Lastly, available data-driven algorithms tend to resemble ’black-box’ approaches, thereby not providing a transparent evaluation of intuitive and clinically established criteria, such as clinimetric properties, which is essential to enhance the clinical integration of assessments^6–8^.

**Fig. 1:**
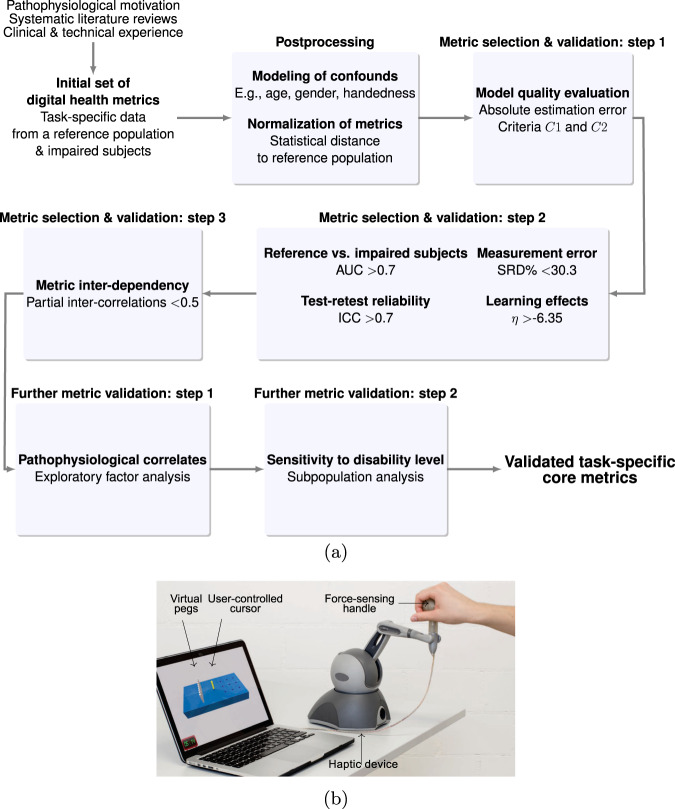
Overview of the metric selection framework and the Virtual Peg Insertion Test (VPIT). **a** The frameworks allows to select a core set of validated digital health metrics through a transparent step-by-step selection procedure. Model quality criteria *C*1 and *C*2; ROC receiver operating characteristics, AUC area under curve, ICC intra-class correlation, SRD% smallest real difference; *η* strength of learning effects. **b** The framework was applied to data recorded with the VPIT, a sensor-based upper limb sensorimotor assessment requiring the coordination of arm and hand movements as well as grip forces.

Hence, the objective of this work was to propose and apply a transparent data-driven framework to select and validate digital health metrics, aimed at providing clinically relevant evidence that facilitates their integration into research trials. The approach (Fig. 1a) relies on (i) a use-case-specific pathophysiological motivation for digital health metrics to represent clinically relevant impairments, considers (ii) the modeling of confounds arising through participant demographics, and implements (iii) data processing steps to quantitatively evaluate metrics based on the most important clinimetric properties (discriminant validity, structural validity, test–retest reliability, measurement error, and learning effects). Herein, we present this framework in the context of a use-case with the Virtual Peg Insertion Test (VPIT, Fig. 1b), an instrumented assessment of upper limb sensorimotor impairments consisting of a goal-directed manipulation task in a virtual environment^34–39^. We hypothesized that the presented methodology would be able to reduce a large set of metrics to a core set with optimal clinimetric properties that allows longitudinally assessing the severity of the targeted impairments in a robust and insightful manner.

Targeting this objective is important, as the proposed data-driven framework can easily be applied to metrics gathered with other digital health technologies. This will help addressing the lacking evaluation, standardization, and interpretability of digital health metrics, a necessary step to improve their still limited clinical relevance^15,22,23^. Further, the presented use-case establishes a validated core set of metrics for the VPIT, paving the way for its integration into clinical trials in neurorehabilitation.

## Results

### Overview of the framework for the selection and validation of digital health metrics

In the following, a summary of the proposed framework is provided (Fig. 1), whereas methodological and implementation details can be found in the Methods. The MATLAB source code for metric selection framework is publicly available at: https://github.com/ChristophKanzler/MetricSelectionFramework.

The framework starts with pathophysiological hypotheses about the connection of the digital health metrics to the impairments that are targeted with a specific technology-aided assessment. Subsequently, the first metric selection step requires that the influence of participant demographics such as age, sex, and tested body side can be accurately compensated through multi-dimensional mixed effect models, as defined by the models’ absolute estimation error (quality criteria *C*1 and *C*2, Eqs. (5) and (6)). As part of the second metric selection step, metrics have to sensitively discriminate between intact and affected subjects as defined by an receiver-operating characteristic (ROC) analysis, thereby providing strong evidence of their ability to identify specific impairments. Additionally, the metrics need to have at least acceptable test–retest reliability as defined by the intra-class correlation coefficient (ICC ≥ 0.7), which allows to longitudinally discriminate across subjects when monitoring recovery. Further, metrics with highest measurement error, as defined by the smallest real difference (SRD% < 30.3) are removed. This ensures that intervention-induced changes can be sensitively captured. Also, metrics with strong learning effects, as defined by the systematic change between test and retest (*η* > −6.35) are discarded to allow a discrimination between task-related learning and intervention-induced changes. As a third step, redundant information are removed via a partial correlation analysis (*ρ*_p_ < 0.5) to foster clinical interpretability and provide a concise set of highly informative metrics. Lastly, two additional validation steps ensure that the metrics are able to capture clinically defined disability levels and enable a speculative discussion of the initially defined pathophysiological hypothesis based on an exploratory factor analysis.

### Application of the framework to the VPIT

In the following, the exemplary use-case of the metric selection framework with the VPIT is presented, whereas an extensive comparative analysis between the proposed framework and three established machine learning-based metric selection algorithms can be found in the supplementary material (Supplementary Fig. 1, Supplementary Tables 1–3).

In more detail, 77 kinematic and kinetic metrics (Tables 2 and 3, see “Methods” section for details) that can be extracted from the VPIT were physiologically motivated by connecting the expected abnormal movement patterns during a goal-directed task to their underlying sensorimotor impairments, using both neuroscience-oriented and clinically-oriented concepts. Subsequently, the framework was applied to VPIT data (Table 1) that were collected in 120 neurologically intact subjects (i.e., normative reference) and 89 neurologically affected subjects (53 with stroke, 28 with MS, and 8 with autosomal recessive spastic ataxia of Charlevoix-Saguenay (ARSACS)). In total, data from 43,350 individual movements were recorded. The neurologically intact subjects were of age 51.1 [34.6, 65.6] years (median [25th, 75th percentile]; 60 male; 107 right hand dominant; 12 with stereo vision deficits) and 60 of them performed a test–retest session (age 48.8 [40.2, 60.2]; 34 male; 48 right hand dominant; time between sessions 5.0 [4.0, 6.5] days). The neurologically affected subjects were 56.2 [42.1, 65.3] years old, 52 were male, 75 were right hand dominant, and for 35 stroke subjects, the right body side was most affected. Most individuals had moderate to mild levels of upper limb disability, which was characterized with conventional scales that are commonly used for each population. The Fugl-Meyer assessment for the upper extremity (FMA-UE) was 57 [49, 65] for post-stroke subjects, the action research arm test (ARAT) was 52.0 [46.5, 56.0] for subjects with MS, and the nine hole peg test (NHPT) was 43.5 [33.1, 58.7] s in subjects with ARSACS. Detailed demographic and clinical information can be found in Supplementary Table 4.

**Table 1:** Demographics and clinical characteristics of the study population.

Characteristics	Unit	Neurologically intact	Stroke	Multiple sclerosis	ARSACS
*N*		120	53	28	8
Age	years	51.1 [34.6, 65.6]	59.0 [52.0, 69.0]	54.5 [39.0, 63.0]	37.0 [30.0 48.5]
Gender	m/f	60/60	37/16	12/16	4/4
FMA-UE	0–66	–	57 [49, 65]	–	
ARAT	0–57	–	–	52.0 [46.5, 56.0]	–
NHPT	s	–	–		43.5 [33.1, 58.7]

Values reported as median [25th, 75th percentile].

*ARSACS* autosomal recessive spastic ataxia of Charlevoix-Saguenay, *FMA-UE* Fugl-Meyer assessment for the upper extremity, *ARAT* action research arm test, *NHPT* nine hole peg test.

### Selection of metrics: step 1

**Fig. 2:**
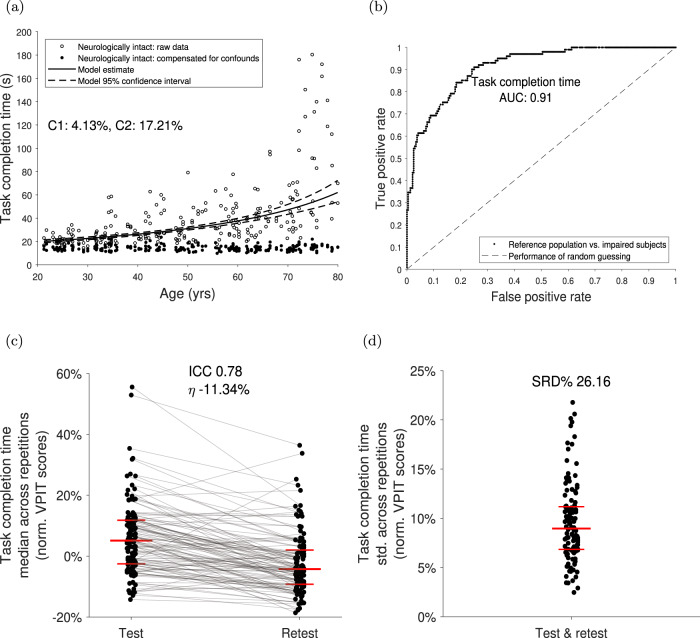
Data-driven selection and validation of metrics: example of task completion time. **a** The influence of age, sex, tested body side, handedness, and stereo vision deficits on each digital health metrics was removed using data from neurologically intact subjects and mixed effect models (model quality criteria *C*1 and *C*2). Models were fitted in a Box–Cox-transformed space and back-transformed for visualization. Metrics with low model quality (*C*1 > 15% or *C*2 > 25%) were removed. **b** The ability of a metric to discriminate between neurologically intact and affected subjects (discriminant validity) was evaluated using the area under the curve value (AUC). Metrics with AUC < 0.7 were removed. **c** Test–retest reliability was evaluated using the intra-class correlation coefficient (ICC) indicating the ability of a metric to discriminate between subjects across testing days. Metrics with ICC < 0.7 were removed. Additionally, metrics with strong learning effects (*η* > −6.35) were removed. The long horizontal red line indicates the median, whereas the short ones represent the 25th and 75th percentile. **d** Measurement error was defined using the smallest real difference (SRD%), indicating a range of values for that the assessment cannot discriminate between measurement error and physiological changes. The distribution of the intra-subject variability was visualized, as it strongly influences the SRD. Metrics with SRD% > 30.3 were removed.

The influence of potential confounds arising from subject demographics and the model quality for each sensor-based metric including *p*-values can be found in Supplementary Table 5 (example in Fig. 2). For all metrics, 69.7%, 44.7%, 27.6%, 6.6%, and 7.9% were significantly (*p* < 0.05) influenced by age, sex, tested side, hand dominance, and stereo vision deficits, respectively. The required quality of the models, according to the *C*1 and *C*2 criteria, were not fulfilled by 13 (16.9%) of all metrics, including a simulated Gaussian noise metric aimed at testing the robustness of the framework.

### Selection of metrics: step 2

Thirteen (16.9%) out of 77 metrics fulfilled the criteria of the validity, reliability, measurement error, and learning analysis (Fig. 2, Tables 2 and 3). The median AUC, ICC, SRD%, and *η* values of the 12 metrics that passed steps 1 and 2 were 0.77 [0.74, 0.85], and 0.80 [0.75, 0.82], 24.6 [21.5, 26.2], and −5.72 [−6.09, −3.27], respectively. The simulated Gaussian noise metric did not pass this evaluation step (AUC = 0.37, ICC = −0.07, SRD% = 117.04, *η* = 0.25).

**Table 2:** Results for the data-driven selection of kinematic metrics.

Movement characteristic	Sensor-based metric	Validity: AUC	Reliability: ICC	Error: SRD%	Learning: *η*
Mov. smoothness TP	Jerk TP	0.80	0.69	23.10	−4.41
	Log jerk TP^a^	0.78	0.74	26.11	−4.82
	SPARC TP^b^	0.84	0.83	23.78	−7.16
	Num of velocity peaks TP^b^	0.82	0.79	21.30	−6.36
	Distance to max. velocity TP^b^	0.44	0.74	33.64	2.42
	Time to max. velocity TP^b^	0.45	0.78	28.70	3.93
Mov. smoothness RT	Jerk RT	0.84	0.68	20.83	−4.70
	Log jerk RT^a^	0.73	0.75	25.33	−6.08
	SPARC RT^a^	0.71	0.76	28.93	−1.57
	Num. velocity peaks RT^a,b^	0.76	0.70	23.27	−3.28
	Distance to max. velocity RT	0.43	0.65	41.39	3.67
	Time to max. velocity RT	0.48	0.73	33.99	2.43
Mov. efficiency TP	Path length ratio TP^a^	0.89	0.76	24.24	−2.17
	Throughput TP^b^	0.92	0.81	24.07	−12.18
Mov. efficiency RT	Path length ratio RT^a^	0.83	0.79	17.30	−3.61
	Throughput RT	0.90	0.78	27.43	−13.21
Mov. curvature TP	Trajectory error mean TP	0.55	0.86	17.14	−0.60
	Trajectory error max. TP	0.57	0.86	15.84	−0.37
	Initial mov. angle TP *θ*_1_^b^	0.67	0.90	13.56	−1.50
	Initial mov. angle TP *θ*_2_^b^	0.67	0.90	13.29	−1.52
	Initial mov. angle TP *θ*_3_	0.61	0.88	14.37	−2.06
Mov. curvature RT	Trajectory error mean RT	0.56	0.84	20.00	1.24
	Trajectory error max. RT	0.55	0.84	18.58	1.22
	Initial mov. angle RT *θ*_1_	0.51	0.75	33.90	3.18
	Initial mov. angle RT *θ*_2_	0.51	0.71	28.65	2.92
	Initial mov. angle RT *θ*_3_	0.60	0.79	23.99	1.53
Mov. speed TP	Velocity mean TP	0.83	0.88	20.61	−9.99
	Velocity max. TP	0.83	0.87	18.57	−9.14
Mov. speed RT	Velocity mean RT	0.75	0.87	19.01	−7.60
	Velocity max. RT^a^	0.76	0.86	19.41	−6.27
Endpoint error peg approach	Position error peg approach	0.86	0.64	29.54	−4.66
	Jerk peg approach^a^	0.74	0.72	27.65	−2.94
	Log jerk peg approach	0.69	0.75	30.20	−8.36
	SPARC peg approach	0.78	0.64	46.55	−10.29
Endpoint error hole approach	Position error hole approach	0.94	0.76	31.29	−5.36
	Jerk hole approach	0.57	0.68	30.63	−4.84
	Log jerk hole approach	0.66	0.83	23.25	−6.53
	SPARC hole approach^a^	0.86	0.81	24.81	−5.72
Haptic collisions TP	Haptic collisions mean TP	0.61	0.85	24.55	−3.99
	Haptic collisions max. TP	0.63	0.84	20.54	−1.08
Haptic collisions RT	Haptic collisions mean RT	0.61	0.72	25.32	−0.07
	Haptic collisions max. RT^b^	0.46	0.79	27.02	4.37
Number of movements	Number of mov. onsets	0.22	0.22	61.34	−0.82
	Number of mov. ends	0.09	0.29	57.01	0.00
Object drops	Number of dropped pegs	0.65	0.50	41.11	−3.20

The area under the curve (AUC, optimum at 1), intraclass correlation coefficient (ICC, optimum at 1), the smallest real difference (SRD%, optimum at 0), and *η* value (optimum at 0, worst at −∞) were used to describe discriminative validity, test–retest reliability, measurement error, and learning effects, respectively.

*mov* movement, *TP* transport, *RT* return, *SPARC* spectral arc length, *num* number.

^a^Metric fulfilled all evaluation criteria (AUC > 0.7, ICC > 0.7, SRD% = −6.35).

^b^Insufficient model quality according to selection step 1.

**Table 3:** Results for the data-driven selection of kinetic metrics.

Movement characteristic	Sensor-based metric	Validity: AUC	Reliability: ICC	Error: SRD%	Learning: *η*
GF scaling TP	GF mean TP	0.40	0.84	14.46	0.39
	GF max. TP	0.40	0.86	15.19	0.07
	GF rate mean TP	0.25	0.87	12.14	2.07
	GF rate max. TP	0.25	0.79	20.53	3.93
GF scaling RT	GF mean RT	0.49	0.76	27.62	0.17
	GF max. RT	0.45	0.66	37.61	2.80
	GF rate mean RT	0.07	0.82	27.79	5.87
	GF rate max. RT	0.29	0.48	34.05	7.19
GF scaling peg approach	GF mean peg approach	0.45	0.83	18.09	1.10
	GF max. peg approach	0.39	0.84	19.40	−0.72
	GF rate mean peg approach	0.18	0.88	14.76	3.54
	GF rate max. peg approach	0.32	0.84	19.52	0.74
GF scaling hole approach	GF mean hole approach	0.36	0.81	15.34	0.76
	GF max. hole approach	0.37	0.82	16.43	0.50
	GF rate mean hole approach	0.15	0.82	14.18	2.73
	GF rate max. hole approach	0.28	0.77	21.41	1.82
GF coord. TP	GF rate num. peaks TP^a^	0.74	0.81	20.59	−6.11
	GF rate SPARC TP^a^	0.74	0.82	22.48	−5.71
GF coord. RT	GF rate num. peaks RT	0.60	0.83	20.17	−4.16
	GF rate SPARC RT	0.64	0.78	23.81	−6.35
GF coord. peg approach	GF rate num. peaks peg approach	0.90	0.78	25.60	−12.25
	GF rate SPARC peg approach	0.90	0.83	22.99	−8.19
GF coord. hole approach	GF rate num. peaks hole approach^a^	0.91	0.81	24.29	−6.14
	GF rate SPARC hole approach^a^	0.84	0.82	26.38	−5.94
GF coord. buildup	GF rate num. peaks buildup^b^	0.15	0.44	57.70	0.77
	GF rate SPARC buildup^b^	0.56	0.79	28.62	−3.22
	GF buildup duration	0.70	0.82	21.36	−6.97
GF coord. release	GF rate num. peaks release^b^	0.44	0.48	56.80	1.78
	GF rate SPARC release	0.91	0.86	18.63	−6.78
	GF release duration	0.67	0.81	21.63	−2.78
Overall disability	Task completion time	0.91	0.78	26.16	−11.34
	Simulated Gaussian noise^b^	0.37	−0.07	117.04	0.25

The area under the curve (AUC, optimum at 1), intraclass correlation coefficient (ICC, optimum at 1), the smallest real difference (SRD%, optimum at 0), and *η* value (optimum at 0, worst at −∞) were used to describe discriminative validity, test–retest reliability, measurement error, and learning effects, respectively. The task completion time and the simulated Gaussian noise metrics were evaluated in addition to the kinetic metrics.

*GF* grip force, *TP* transport, *RT* return, *SPARC* spectral arc length, *num* number.

^a^Metric fulfilled all evaluation criteria (AUC > 0.7, ICC > 0.7, SRD% = −6.35).

^b^insufficient model quality according to selection step 1.

### Selection of metrics: step 3

**Fig. 3:**
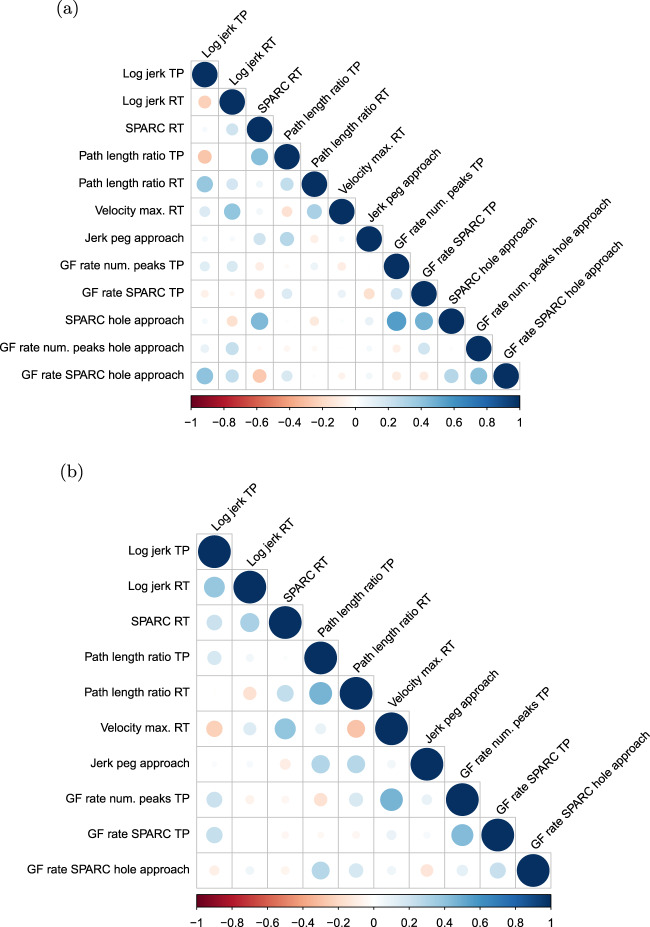
Partial correlation analysis. The objective was to remove redundant information. Therefore, partial Spearman correlations were calculated between all combination of metrics while controlling for the potential influence of all other metrics. Pairs of metrics were considered for removal if the correlation was equal or above 0.5 The process was done in an iterative manner and the first **a** and the last **b** iterations are presented.

The constructed partial correlation matrices can be found in Fig. 3. Among the remaining metrics, grip force rate number of peaks hole approach was removed as it correlated (*ρ*_p_ ≥ 0.5) with grip force rate spectral arc length approach hole and the latter metric is less influenced through confounds as it is independent of movement distance. Additionally, spectral arc length hole approach was discarded as it correlated with grip force rate spectral arc length hole approach and the latter metric is more directly related to hand function, which was not yet well covered by the other metrics. The remaining 10 metrics yielded absolute partial inter-correlations of 0.14 [0.06 0.24] (zero very high, zero high, zero moderate, six low, and 39 very low inter-correlations).

### Further validation of metrics: step 1

The Kaiser–Meyer–Olkin value was 0.82, which indicated that the application of the factor analysis was suitable^40,41^. According to the parallel analysis, the most likely number of underlying latent factors *k* was five (Supplementary Fig. 3). The factor loadings can be found in Table 4. The metrics path length ratio transport/return and jerk peg approach had strong loadings on factor 1. The metrics log jerk transport, log jerk return, and spectral arc length return loaded strongly on factor 2. The metrics grip force rate number of peaks transport and grip force rate spectral arc length transport had strong loadings on factor 3, whereas velocity max. return and grip force rate spectral arc length hole approach loaded strongly on factors 4 and 5, respectively.

**Table 4:** Structural validity: exploratory factor analysis.

Expected interpretation	Sensor-based metric	F1	F2	F3	F4	F5
Movement smoothness transport	Log jerk transport	0.09	0.73^a^	0.21	−0.19	−0.05
Movement smoothness return	Log jerk return	−0.08	0.86^a^	−0.11	0.02	0.02
	SPARC return	0.10	0.59^a^	−0.10	0.23	−0.03
Movement efficiency transport	Path length ratio transport	0.83^a^	0.08	−0.17	0.06	0.11
Movement efficiency return	Path length ratio return	0.79^a^	−0.06	0.08	−0.14	0.04
Movement speed transport	Velocity max. return	−0.02	0.01	0.16	0.90^a^	0.01
Endpoint error peg approach	Jerk peg approach	0.72^a^	−0.04	0.12	0.07	−0.14
GF coord. transport	GF num. peaks transport	0.00	−0.06	0.93^a^	0.11	−0.03
	GF rate SPARC transport	−0.08	0.19	0.62^a^	0.00	0.11
GF coord. hole approach	GF rate SPARC hole approach	0.11	−0.02	0.02	0.01	0.94^a^

Loadings of metrics on underlying latent factors extracted with exploratory factor analysis. The interpretation of each metric was physiologically motivated initially. Larger absolute loadings indicate a stronger contribution to a factor.

*F1–5* data-driven latent factors, *GF* grip force, *coord* coordination, *num* number, *SPARC* spectral arc length.

^a^Indicates strong loadings (i.e., absolute loading of at least 0.5).

### Further validation of metrics: step 2

**Fig. 4:**
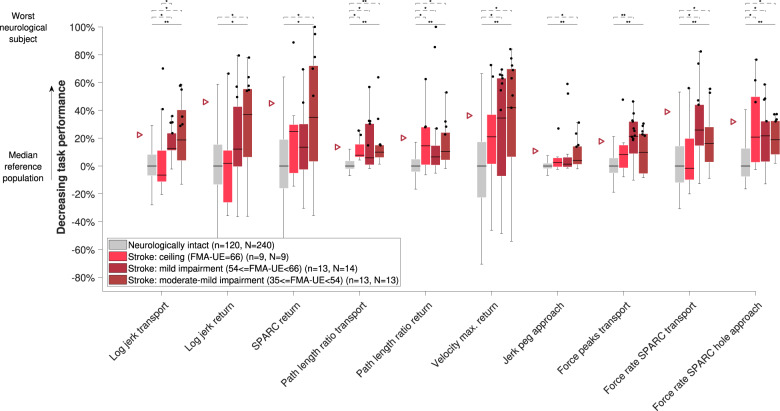
Sensitivity of metrics to disability severity in stroke subjects. Subjects were grouped according to the clinical disability level. The vertical axis indicates task performance based on the distance to the reference population. The population median is visualized through the black horizontal line, the interquartile range (IQR) through the boxes, and the min and max value within 1.5 IQR of the lower and upper quartiles, respectively, through the whiskers. Data points above the 95th-percentile (triangles) of neurologically intact subjects are showing abnormal behavior (black dots). Solid and dashed horizontal black lines above the box plots indicate results of the omnibus and post-hoc statistical tests, respectively. *Indicates *p* < 0.05 and **p < 0.001. *n* refers to the number of subjects in that group and *N* to the number of data points. Only subjects with available clinical scores were included. For the jerk peg approach, one outlier was not visualized to maintain a meaningful representation. FMA-UE Fugl-Meyer upper extremity, SPARC spectral arc length.

**Fig. 5:**
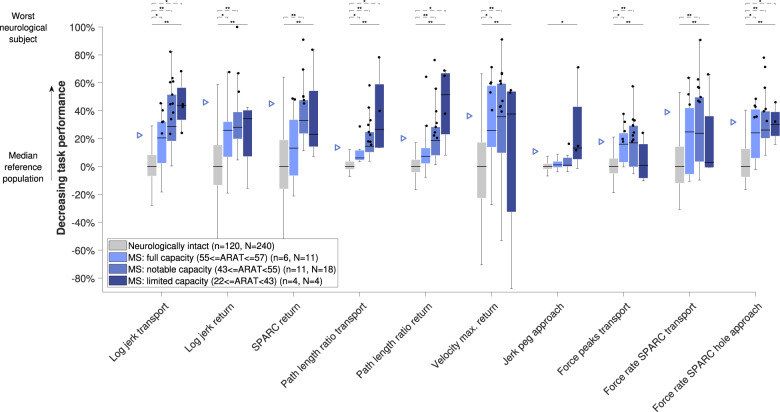
Sensitivity of metrics to disability severity in MS subjects. See Fig. 4 for a detailed description. ARAT action research arm test.

**Fig. 6:**
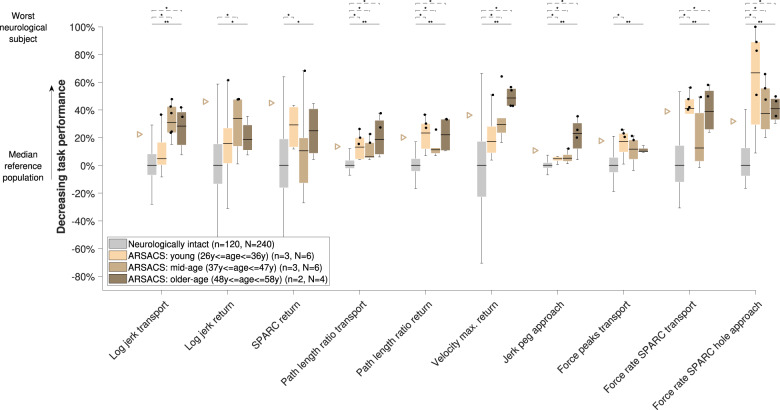
Sensitivity of metrics to disability severity in ARSACS subjects. See Fig. 4 for a detailed description.

The behavior of all metrics across subject subpopulations with increasing disability level can be found in Figs. 4–6. All metrics indicated statistically significant differences between the neurologically intact and at least one of the neurologically affected subpopulations for each disorder, with the exception of jerk peg approach in MS subjects (omnibus *p* = 0.001, three between-groups degrees of freedom (DoF), *H* = 17.3, post-hoc *p* > 0.05). Additionally, significant differences between subpopulations were found for log jerk transport in stroke subjects (omnibus *p* < 0.001, three between-groups DoF, *H* = 25.3, post-hoc *p* = 0.024). Consistent trends (i.e., monotonically increasing medians across subpopulations) were found for all metrics except for spectral arc length return, force rate spectral arc length approach hole, and force rate num. peaks approach hole.

## Discussion

In this work, we aimed to propose and apply a transparent data-driven framework to select and validate digital health metrics, with the objective to provide clinically relevant evidence that facilitates their still lacking clinical integration. The framework considers (i) the targeted impairments, (ii) the influence of participant demographics, and (iii) important clinimetric properties. As an example use-case, we implemented this framework with 77 kinematic and kinetic metrics extracted from the VPIT, a previously proposed sensor-based assessment of arm and hand sensorimotor impairments. For this purpose, the VPIT was administered to 120 neurologically intact and 89 neurologically affected subjects, yielding data from 43.350 individual movements.

This objective methodology to identify a core set of validated metrics based on pathophysiological hypotheses and quantitative selection criteria can complement currently applied paradigms for selecting digital health metrics^21,27–31,42^. While consensus-based recommendations from groups of experts are indispensable for constructing high-level hypothesis (e.g., which body functions to assess in a given context), the selection of specific sensor-based metrics should solely be implemented based on objective and data-driven evaluation criteria to avoid selection bias. Also, guidelines to pool data within systematic reviews, often intended for the selection of conventional assessments, need to be considered carefully in the context of digital health metrics. Compared to conventional assessments that often provide a single, intuitively understandable, task-specific metric (e.g., FMA-UE score), a plethora of abstract digital health metrics exists and the same metric (e.g., log jerk) can be extracted from all technologies sharing similar sensor data. However, for a meaningful interpretation of sensor-based metrics, it is essential to consider them in light of the assessment context, as data processing steps (e.g., filter design), assessment platform type (e.g., end-effector or camera-based system), task type (e.g., goal-directed or explorative movements), and target population (e.g, neurological or musculoskeletal impairments) strongly influences the anticipated hypotheses and clinimetric properties^13^. This emphasizes the importance of a validation and selection of each metric in its specific context (i.e., assessment platform, task, and target population), which can hardly be achieved when relying on consensus-based or review-based approaches. While data-driven, context-specific metric selection algorithms leveraging on the nowadays existing big data sets are well established in the machine-learning domain (therein referred to as feature selection algorithms), these typically attempt to reconstruct accurate ground truth information about the targeted impairment (supervised learning) by combining multiple predictors in a mathematical model^31–33,43^. However, the metrics selected by such models might only carry insightful information in combination with other metrics^43^, thereby challenging the use of individual metrics as clinical endpoints, as visible in Supplementary Tables 1–3. In addition, a gold standard is unfortunately often not available in certain healthcare domains, as for example knowledge about the history of neurological injury does not directly represent a ground truth for the severity of specific sensorimotor impairments. Hence, novel algorithms are required that can achieve a robust selection of metrics with inaccurate ground truth (weakly supervised learning)^44^. Further, while existing feature selection algorithms typically yield optimal solutions in terms of a mathematical loss function, they are often not providing a transparent evaluation with evidence that can be easily interpreted by healthcare practitioners and do not necessarily select metrics that fullfil all clinimetric properties (Supplementary Tables 1–3). This, however, is fundamental for paving the way for the clinical acceptance of novel assessments^6–8^. The proposed approach attempts to address these challenges, by enabling a robust selection of individual metrics with inaccurate ground truth (weakly supervised learning), by providing a transparent evaluation based on a step-by-step procedure, and by creating a foundation of clinically relevant evidence about the quality of the assessment. This creates an interesting alternative for researchers in the field of digital health to more established feature selection algorithms, which are not optimized for the unique requirements of digital health metrics. Ultimately, this might help to better transfer research findings into clinical healthcare environments^15,22^.

For accurate comparisons between neurologically intact and affected subjects, it is essential to account for the difference in potential confounds, such as demographical characteristics, between the groups. The presented analysis adds an important methodological contribution to previous work that used linear models to compensate for confounds by additionally evaluating the quality of these models^45–48^. This allowed to discard metrics for which the confounds could not be accurately modeled (16.8% of all metrics). Especially metrics that have mathematical support with two finite boundaries (e.g., 0% and 100%) received low model quality, which can result from skewness and heteroscedasticity that cannot be corrected using variance-stabilizing transformations, such as the Box–Cox method. Such metrics should therefore be considered carefully and other modeling approaches, for example based on beta distributions, might be required to accurately compensate for the effect of measurement confounds^49^. Eighty-three percent of all metrics (Tables 2 and 3) were discarded through the second selection step. It is fundamental to understand that these evaluation criteria (validity: AUC, reliability: ICC, measurement error: SRD%, learning effects: *η*) are complementary to each other, focusing on different components of intra-subject and inter-subject variability, which are all essential to sensitively monitor impairments. It is therefore not sufficient to solely consider a subset of these criteria, as often done in literature. Evaluating the validity of sensor-based metrics using a reference population and ROC analysis is superior to the more commonly applied correlations with conventional scales (concurrent validity)^24,25^. A reason for this is that digital health metrics are often expected to provide complementary information to conventional scales that improves upon their limitations, thereby challenging the definition of accurate hypothesis about the correlation between conventional and sensor-based scales. Nevertheless, comparisons between metrics and conventional scales can help to better interpret sensor-based metrics or to test their sensitivity to impairment severity, as attempted in the last validation step. This analysis was not used as a criteria for metric selection as, to expect trends across subgroups, each sensor-based metric would require a carefully selected clinical counterpart that captures a similar physiological construct. Also, stepwise regression approaches that model conventional scales in order to select metrics have been extensively applied even though they have been considered bad practice due to statistical shortcomings^50–53^. Lastly, a simulated metric without relevant information content (simulated Gaussian noise) was rejected in the first and second selection steps, thereby providing evidence that the framework allows to discard certain physiologically irrelevant metrics.

Applying the proposed framework, 10 almost independent metrics (Table 4) were identified as a validated core set for the VPIT and were able to reliably assess the severity of multiple sensorimotor impairments in arm and hand for subjects with mild to moderate disability levels (i.e., the target population of the VPIT). These metrics were related to the movement characteristics smoothness, efficiency, speed, endpoint error, and grip force coordination during specific phases of the task (gross movements transport and return; fine movements peg approach, and hole approach). While these characteristics are generally expected to inform on abnormal feedforward control, impaired somatosensory feedback, increased muscle tone, abnormal flexor synergies, dysmetria, and weakness, the clustering of the metrics into five factors allows to further speculate about their interpretation (Table 4). The first factor was dominated by movement efficiency metrics (path length ratio transport and return), and the jerk peg approach as a descriptor for the endpoint error of a movement, thereby informing on the speed-accuracy tradeoff that is a typical characteristic of goal-directed movements^54,55^. The second factor contained metrics focusing on movement quality (smoothness) during transport and return, which is expected to describe impaired feedforward control of arm movements. Hence, it is unlikely that the first factor also informs on feedforward control. We therefore expect the movement efficiency metrics (first factor) to be rather related to flexor synergy patterns, weakness, proprioceptive deficits, and dysmetria. Among these impairments, weakness and proprioceptive deficits are most commonly observed in neurological disorders^2,56^. The third factor focused on grip force coordination during transport (grip force rate num. peaks transport and grip force rate spectral arc length transport), which is expected to be related to abnormal feedforward control and impaired somatosensory feedback. The dissociation between factor one and three is interesting, as it suggests different control schemes underlying the regulation of arm movements and grip forces. A tight predictive coupling between the modulation of grip forces and rapid arm movements has been reported in neurologically intact subjects^57^. The factor analysis suggests that this predictive coupling might possibly be disrupted in neurologically affected subjects, potentially due to altered sensory feedback (e.g., proprioception) leading to inaccurate predictive internal models or abnormal neural transmission (e.g., corticospinal tract integrity)^58,59^. Reduced corticospinal tract integrity can also lead to weakness and could affect movement speed, as described by factor four (velocity max. return)^58^. This factor might further be influenced by an altered inhibition of the supraspinal pathways, often resulting from upper motor neuron lesions, leading to increased muscle tone and thereby altered movement speed^60^. Lastly, the fifth factor covered grip force coordination during hole approach, thereby diverging from the coordination of grip forces during gross movements (transport) as described by factor 3 and focusing more on grip force coordination during precise position adjustments. This suggests that the two phases are differently controlled, potentially because the hole approach is more dominated by sensory and cognitive feedback loops guiding the precise insertion of the peg, whereas gross movements (transport) are more dominated through feedforward mechanisms^59^. Also, the physiological control origin of the two movement phases might differ, as gross movements are expected to be orchestrated by the reticulospinal tract, whereas precise control is more linked to the corticospinal tract^61^. Even though the task completion time did not pass the selection procedure due to strong learning effects, one might still consider to report the metric when using the VPIT in a cross-sectional manner as its intuitive interpretation allows to give an insightful first indication about the overall level of impairment that might potentially be interesting for both clinical personnel and the tested patient.

The added clinical value of the VPIT core metrics compared to existing conventional assessments is visible in Figs. 4 and 5, as the former allowed to detect sensorimotor impairments in certain subjects that did not show any deficits according to the typically used conventional scales. Such a sensitive identification of sensorimotor impairments might allow to provide evidence for the potential of additional neurorehabilitation. Further, the identified core set of metrics can efficiently inform on multiple impairments, both sensory and motor, in arm and hand with a single task that can typically be performed within 15 min per upper limb. This advances the state-of-the-art that mainly focused on the evaluation of arm movements^18,62,63^, or required more complex or time-consuming measurement setups (e.g., optical motion capture) to quantify arm and hand movements while also neglecting grasping function^64^. Such a fine-grained evaluation covering multiple sensorimotor impairments can help to stratify subjects into homogeneous groups with low inter-subject variability. This is important to reduce the required number of subjects to demonstrate significant effects of novel therapies in clinical trials^18^. To further complement such clinic-bound assessments, wearable sensors could help to passively monitor individuals with higher time-resolution, thereby allowing to better capture the impact of interventions on daily life participation^65^. In such scenarios, it is likely that the selection of clinically relevant core metrics from wearable sensor data would also benefit from the proposed metric selection framework.

The developed methodology should be considered in light of certain limitations. Most importantly, the framework was especially designed for metrics aimed at longitudinally monitoring impairments and might need additional refinement when transferring it to other healthcare applications, such as screening of electronic health record data, with different clinical requirements. Hence, in the future, the applicability of the framework to other data types and applications should be explored. Also, while the framework seems optimal for digital health metrics aimed at repeatedly assessing impairments, it might not be ideal in scenarios where the defined clinimetric properties are not the main clinically relevant criteria. In such cases, mathematically optimal methods such as LASSO might prove more versatile. Additionally, the definition of multiple cut-off values for the metric selection process influences the final core set of metrics. Even though most of the cut-offs were based on accepted definitions from the research community (e.g., COSMIN guidelines), we acknowledge that the optimality of these values needs to be further validated from a clinical point of view. To evaluate measurement error and learning effects, novel cut-offs were introduced based on the distribution of observed values for the VPIT with the goal to exclude metrics that showed highest measurement error and strongest learning effects. It is important to note that this only considers the relative and not the absolute level of measurement error. However, this can only be adequately judged using data recorded pre-intervention and post-intervention, allowing to compare the measurement error (SRD%) to intervention-induced physiological changes (minimal important clinical difference)^25^. Hence, the rather high absolute level of observed measurement errors for the VPIT (up to 57.7% of the range of observed values) warrants further critical evaluation with longitudinal data. Also, it is important to note that, even though certain metrics did not pass the selection procedure, they might still prove to be valid and reliable for other assessment tasks and platforms, or more specific subject populations. In this context, it should be stressed that test–retest reliability, measurement error, and learning effects for the metrics were evaluated with neurologically intact subjects and might require additional investigation in neurological populations. Regarding the VPIT, the effect of the virtual reality environment on the extracted metrics should be thoroughly characterized in the future^66,67^.

In conclusion, we proposed a transparent, weakly supervised, and data-driven framework for selecting and validating digital health metrics based on the targeted impairments, the influence of participant demographics, and clinimetric properties. This framework can complement existing feature selection algorithms that are mathematically optimal, but are less transparent and require accurate ground truth. In a use-case with the VPIT, the methodology enabled the selection and validation of a core set of 10 kinematic and kinetic metrics out of 77 initially proposed metrics. The chosen metrics were able to accurately describe the severity of multiple sensorimotor impairments in a cross-sectional manner and have high potential to sensitively monitor neurorehabilitation and to individualize interventions. Additionally, an in-depth physiological motivation of these metrics and the interpretation based on an exploratory factor analysis allowed to better understand their relation to the targeted impairments. Hence, this work makes an important contribution to implement digital health metrics as complementary endpoints for clinical trials and routine, next to the still more established conventional scales and patient reported metrics^68^. We urge researchers and clinicians to capitalize on the promising properties of digital health metrics and further contribute to their validation and acceptance, which in the long-term will lead to a more thorough understanding of disease mechanisms and enable novel applications, such as a personalized predictions of therapy outcomes, with the potential to improve healthcare quality.

## Methods

To objectively reduce a large set of digital health metrics to a clinically relevant subset, we implemented a three-step process (Fig. 1) considering the most important statistical requirements to sensitively and robustly monitor impairments in a longitudinal manner. These requirements were inspired from the COSMIN guidelines for judging the quality of metrics based on systematic reviews and related work on digital health metrics^13,25,42,45,69^. Further, two additional validation steps were implemented to improve the understanding of the selected core metrics (Fig. 1). While this selection and validation framework is independent of a specific assessment platform (i.e., the initial set of metrics to be evaluated), the manuscript defines the framework in the context of the VPIT with the goal to provide specific instructions including a hands-on example, starting from the initial motivation of metrics to the selection of a validated core set. This work was previously published in pre-print form^70^.

### Virtual Peg Insertion Test

The VPIT is a digital health assessment combining a commercial haptic end-effector (PHANTOM Omni/Touch, 3D Systems, CA, USA), a custom-made handle with piezoresistive force sensors (CentoNewton40, Pewatron AG, Switzerland), and a virtual reality (VR) environment, implemented in C++ and OpenGL on a Microsoft (Redmond, WA, USA) Windows laptop (Fig. 1). The assessment features a goal-directed pick-and-place task that requires arm and hand movements while actively lifting the arm against gravity, thereby combining elements of the NHPT and the Box and Block Test^71,72^. The VR environment displays a rectangular board with nine cylindrical pegs and nine corresponding holes arranged as a 3 × 3 matrix with similar dimensions as the NHPT (26.8 × 12.8 × 6.2 cm)^71^. The objective is to transport the virtual pegs into the holes by controlling a cursor through the haptic device, which has six degrees of freedom (three DoF translational movement and three DoF angular orientation). The device can provide haptic feedback about the virtual pegboard of up to 3.3 N on the three translational DoF, while the three rotational DoF are passive. A peg can be picked up by aligning the position of a cursor with the peg (alignment tolerance: 3.0 mm) and applying a grasping force above a 2 N threshold. The peg needs to be transported towards a hole while maintaining a grasping force of at least 2 N, and can be inserted in the hole by releasing the force below the threshold, once properly aligned with a hole. The holes in the board of the VR environment are rendered through reduced haptic impedance compared to other parts of the board. The pegs cannot be picked up anymore upon insertion in a hole and are perceived as transparent throughout the test (i.e., no collisions between pegs are possible). The default color of the cursor is yellow and changes after spatially aligning cursor and peg (orange), during the lifting of a peg (green), or after applying a grasping force above the threshold while not being spatially aligned with the peg (red). During the execution of the task, 6 DoF end-effector movements, grasping forces, and interaction forces with the VR environment are recorded at 1 kHz.

### Participants and procedures

The analysis presented in this work builds on data from different studies that included assessments with the VPIT^35,73–75^. Age-matched reference data was based on 120 neurologically intact subjects. Their handedness was evaluated using the Edinburgh Handedness Inventory and potential stereo vision deficits that might influence the perception of a virtual environment were screened using the Lang stereo test^76^. Sixty of these subjects were further tested a second time one to three days apart to evaluate test-retest reliability. Additionally, 53 post-stroke subjects, 28 MS subjects, and 8 subjects with ARSACS were tested. Each subject was tested with the VPIT on both body sides if possible. The administered conventional assessments were dependent on the disease and the specific study. Commonly applied assessments were the FMA-UE^9^, the NHPT^71^, and the ARAT^77^. Detailed exclusion criteria are listed in the supplementary methods. All subjects gave written informed consent prior to participation in the experiments. All experimental procedures were approved by the following Ethics Committees: neurologically intact subjects subjects EK2010-N-40 at ETH Zurich; stroke subjects EKNZ-2016-02075 at Ethikkommision Nordwest- und Zentralschweiz, KEK-ZH 2011-0268 at Kantonale Ethikkommission Zurich; MS subjects: CME2013/314 at Hasselt University, ML9521 (S55614) at KU Leuven, B322201318078 as Belgian reference number; ARSACS subjects: 2012-012 at CIUSSS Chicoutimi.

To perform the VPIT, participants were seated in a chair with backrest and without armrests in front of a laptop with the haptic device being placed on the side of the tested limb. The initial position of the subjects (i.e., hand resting on the handle) was defined by a shoulder abduction angle of ≈45^∘^, a shoulder flexion angle of ≈10^∘^, and an elbow flexion angle of ≈90^∘^. Subjects received standardized instructions, were allowed to insert all nine pegs once for familiarization with the task and virtual environment (data not analyzed), and subsequently performed five repetitions (i.e., inserting all nine pegs five times) per body side. Participants were instructed to perform the task as fast and accurately as possible, and received live feedback about their task completion time via a timer.

### Data preprocessing

Data preprocessing steps are required to optimize the quality of the sensor data and dissect the complex recorded movement patterns into distinct movement phases that can be related to specific sensorimotor impairments. All data preprocessing steps are explained in detail in the Supplementary material and only a brief overview is provided in the following. First, temporal gaps lager than 50 samples in the sensor data were linearly interpolated. Subsequently, a 1D distance trajectory *d*(*t*) was estimated from the 3D cartesian position trajectories, and velocity (first time-derivative) and jerk (third time-derivative) signals were derived from *d*(*t*). All time-series were low-pass filtered initially and after each derivation (Butterworth filter, fourth order, cut-off frequency 8 Hz).

Subsequently, specific phases of the test were segmented to allow better pinpointing specific sensorimotor impairments (details in Supplementary Methods and Supplementary Fig. 2). The detection of all movement phases relied on a signal recorded by the test indicating the specific peg that is currently lifted and a threshold-based procedure for determining movement start and end. To isolate rapid ballistic movements, the trajectories of each peg were segmented into the transport (i.e., ballistic movement while transporting the peg to a hole) and return (i.e., ballistic movement while returning the cursor to the next peg) phases. To capture the overshoot when reaching for a target as well as the precise position adjustments related to virtual object manipulations, the trajectories were additionally segmented into the peg approach and hole approach phases. The former was defined from the end of the return until the next peg was picked up. The latter was defined from the end of the transport until the current peg was inserted into a hole. Further, grasping forces were additionally segmented into the force buildup (i.e., behavior during the most rapid production of force) and force release phases (i.e., behavior during the most rapid release of force), by first identifying the position of the maximum and minimum value in grip force rate between approaching and inserting each peg.

### Pathophysiological motivation of digital health metrics

To facilitate the pathophysiological interpretation of sensor-based metrics for each use-case, it is of importance to describe the mechanisms underlying a specific disease, their effect on the assessed behavioral construct, and how metrics are expected to capture these abnormalities. Within the use-case of the VPIT, this pathophysiological motivation is implemented using the computation, anatomy, and physiology model, as well as the clinical syndromes ataxia and paresis that are commonly present in neurological disorders^58,78^. Leveraging these concepts allows to especially connect how inappropriately scaled motor commands and an inability to voluntarily activate spinal motor neurons affect upper limb movement behavior. As the VPIT strives to capture multiple heterogeneous and clinically relevant sensorimotor deficits, a variety of different movement characteristics were defined to describe commonly observed upper limb sensorimotor impairments in neurological disorders. Subsequently, an initial set of 77 metrics (Tables 2 and 3) for the VPIT were proposed with the aim to describe these movement characteristics and the associated sensorimotor impairments. These metrics were preselected based on the available sensor data (i.e., end-effector kinematic, kinetics, and haptic interactions), recent systematic literature reviews as well as evidence-based recommendations^13,24,79^, and the technical and clinical experience of the authors.

#### Movement smoothness

Goal-directed movements are executed by translating parameters such as target distance into neural commands of certain amplitude, which are transferred to peripheral muscles performing a movement^59^. The signals’ amplitudes might be chosen to minimize movement endpoint variance, which leads to smooth behavior (i.e., bell-shaped velocity trajectories)^55^. These velocity trajectories can be modeled using a superposition of submovements and minimize the magnitude of the jerk trajectory^80^. In neurological subjects, more submovements with increased temporal shift and higher jerk magnitudes have been observed^81,82^, potentially due to disrupted feedforward control mechanisms. The temporal shift between subcomponents and the jerk magnitude was shown to reduce after receiving rehabilitation therapy^81^, thereby highlighting their relevance to track recovery. We used the integrated jerk (referred to as jerk) normalized with respect to movement duration and length leading to a dimensionless metric to represent the intrinsic minimization of jerk^81^. The same metric was used with an additionally applied transformation (log jerk)^83^. Additionally, the spectral arc length (i.e., metric describing spectral energy content) of the velocity trajectory should reflect the energy induced by jerky movements^83,84^. Further, the number of peaks in the velocity profile (number of velocity peaks; MATLAB function findpeaks) was established as an indicator for the number of submovements. Lastly, we calculated the time (time to max. velocity) and distance (distance to max. velocity) covered at peak velocity normalized with respect to the totally covered distance and time, respectively, to capture deviation from the typically observed bell-shaped velocity profile^63^. We calculated these metrics separately for transport and return as the transport requires precise grip force control, which could further affect feedforward control mechanisms.

#### Movement efficiency

Ballistic movements in healthy subjects tend to follow a trajectory similar to the shortest path between start and target^85^. Previous studies suggested that neurologically affected subjects instead perform movements less close to the optimal trajectory compared to healthy controls^86^ and that this behavior correlates with impairment severity, as measured by the FMA-UE^87^. This suboptimal movement efficiency results in general from abnormal sensorimotor control, for example due to from erroneous state estimates for feedforward control, abnormal muscle synergy patterns (e.g., during shoulder flexion and abduction), weakness, and missing proprioceptive cues^58,86,88^. We used the path length ratio (i.e., shortest possible distance divided by the actually covered distance) to represent inefficient movements^86^. Additionally, the throughput (ratio of target distance and target width divided by movement time) was used as an information theory-driven descriptor of movement efficiency^54,89^. The metrics were extracted from the start of the transport phase until the current peg was released and from the start of the return phase until the next peg was taken, as not only ballistic movements but also the endpoint error is of interest when describing the efficiency of movements.

#### Movement curvature

While movement efficiency describes the overall deviation from the shortest path, it does not account for the direction of the spatial deviation. This might, however, be relevant to better discriminate abnormal feedfoward control from flexor synergy pattern or weakness, as in the latter two cases the movements might be especially performed closer to the body. We therefore selected five additional metrics to analyze the spatial deviation from the optimal trajectory in the horizontal plane^36,37^. The initial movement angle was defined as the angular deviation between the actual and optimal trajectory^88^. As this metric requires the definition of a specific timepoint in the trajectory to measure the deviation, and as multiple approaches were used in literature^63,88–90^, we explored three different ways to define the timepoint. This included the time at which 20% of the shortest distance between peg and hole was covered (initial movement angle *θ*_1_), the time at which 20% of the actually covered distance between peg and hole was reached initial movement angle *θ*_2_, and the time at which peak velocity was achieved (initial movement angle *θ*_3_). Additionally, the mean and maximal trajectory error with respect to the ideal, straight trajectory were calculated. All metrics were estimated separately for transport and return.

#### Movement speed

The speed of ballistic movements in healthy subjects is mostly controlled by the tradeoff between speed and accuracy as described by Fitt’s law, which is indirectly imposed through the concept of velocity-dependent neural noise^54,55^. In neurologically affected subjects, increased speed can, for example, result from inappropriately scaled motor commands and disrupted feedforward control^58^. On the other hand, reduced speed can also stem from weakness (i.e., reduced ability to active spinal motor neurons leading to decreased strength) or spasticity (i.e., velocity-dependent increase in muscle tone), the latter resulting from upper motor neuron lesions, abnormally modulated activity in the supraspinal pathways, and thereby increased hyperexcitability of stretch reflexes^58,60^. We calculated the mean (velocity mean) and maximum (velocity max.) values of the velocity trajectory to represent movement speed during the transport and return phases.

#### Endpoint error

To fully characterize the speed-accuracy tradeoff, we additionally analyzed the position error at the end of a movement. In neurological disorders, increased endpoint error (i.e., dysmetria) was commonly observed and can, for example, result from inappropriately scaled motor commands and thereby disrupted feedforward control^91,92^, but also from cognitive and proprioceptive deficits^93^. Dysmetria was found especially in post-stroke subjects with lateral-posterior thalamic lesions^93^, is a common manifestation of intention tremor in MS^94^, and is typically observed in subjects with cerebellar ataxia^95^. In the VPIT, the horizontal Euclidean distance between the cursor position and targeted peg or hole (position error) was calculated for each sample of the peg approach and hole approach phases, respectively, and summed up across all samples of the phase. Further, the jerk, log jerk, and spectral arc length metrics were calculated during both phases, as a jerk index was shown previously to correlate with the severity of intention tremor in MS^96^.

#### Haptic collisions

Haptic collisions describe the interaction forces between a subject and the virtual pegboard rendered through the haptic device. Haptic guidance can be used to ease inserting the virtual pegs into the holes, which have reduced haptic impedance. Previous studies indicated increased haptic collision forces in multiple neurological disorders and especially stroke subjects with sensory deficits^34,97^. We additionally expected that collision forces during transport and return (i.e., phases during which haptic guidance is not required) could be increased due to arm weakness. In particular, neurological subjects can have a limited capability to lift their arm against gravity, leading to increased vertical haptic collisions^98^. The mean and max. vertical collision force (haptic collisions mean and haptic collisions max.) was calculated during transport and return to quantify haptic collision behavior.

#### Number of successful movements

Subjects without neurological deficits can start and end goal-directed movements with ease. On the contrary, persons with neurological disorders can have a reduced ability to initiate and terminate ballistic movements with potentially heterogeneous underlying impairments including abnormal feedforward control, sensory feedback, spasticity, weakness, and fatigue^13,58,63^. Therefore, the metric number of movement onsets was defined based on the number of valid pegs, using the defined segmentation algorithm, when identifying the start of the transport and return phases. Analogously, number of movement ends was based on the sum of correctly segmented ends for the transport and return phases.

#### Object drops

Neurologically intact subjects can precisely coordinate arm movements and finger forces to transport objects. This ability can be reduced in neurological disorders and can potentially lead to the drop of an object during its transport^99^. Underlying mechanisms include for example distorted force control due to incorrectly scaled motor commands or distorted sensory feedback as well as reduced spatio-temporal coordination between arm and hand movements^58,99^. In the VPIT, the number of virtual pegs that were dropped (dropped pegs) should represent object drops and thereby grip force control as well as the spatio-temporal coordination of arm and hand movements. The metric was defined based on how often the grasping force dropped below a 2 N threshold (i.e., subjects still holding the handle) while lifting a virtual peg ^37^.

#### Grip force scaling and coordination

The precise scaling and spatio-temporal coordination of grasping forces is a key requirement for successful object manipulation and leads, in neurologically intact subjects, to single-peaked bell-shaped grip force rate profiles when starting to grasp objects^100^. Abnormal grip force scaling and decreased grip force coordination have been reported in neurological subjects, resulting in multi-peaked grip force rate profiles, and were attributed to, for example, distorted feedforward control, abnormal somatosensory feedback and processing, as well as the presence of the pathological flexor synergy^100–107^. Also, a reduction in applied grip force levels due to weakness can be expected depending on the neurological profile of a subject^58^. Further, a slowness of force buildup^102^ and force release^103^ has been reported, even though other studies showed that the ability to produce and maintain submaximal grip forces was preserved^99,103^. Additionally, there is evidence suggesting that force buildup and force release have different neural mechanisms and that force control can further be decomposed into force scaling and motor coordination^103,104^.

To describe grip force scaling, we applied four metrics separately to the transport, return, peg approach, and hole approach phases. We calculated the mean (grip force mean) and maximum (grip force max.) value of the grasping force signal during each phase. Additionally, we estimated the mean absolute value (grip force rate mean) and absolute maximum (grip force rate max.) of the grip force rate time-series. Similarly, we characterized grip force coordination during the transport, return, peg approach, hole approach, force buildup and force release phases, for which we calculated the number of positive and negative extrema (grip force rate number of peaks) and the spectral arc length (grip force rate spectral arc length). For the force buildup and force release phases, which contain only the segments of most rapid force generation and release, respectively, we additionally calculated their duration (force buildup/release duration).

#### Overall disability

A single indicator expected to describe the subject-specific overall disability level was defined based on the task completion time (i.e., duration from first transport phase until insertion of last peg).

### Data postprocessing

To reduce the influence of intra-subject variability, the grand median across pegs and repetitions was computed for each metric. Subsequently, the influence of possible confounds, which emerge from subject demographics not related to neurological disorders, was modeled based on data from all neurologically intact subjects. This should allow to compensate for these factors when analyzing data from neurologically affected subjects. In more detail, the impact of age (in years), sex (male or female), tested body side (left or right), and handedness (performing the test with the dominant side: true or false) were used as fixed effects (i.e., one model slope parameter per independent variable) in a linear mixed effect model generated for each sensor-based metric^108^. Additionally, the presence of stereo vision deficits (true or false) was used as a fixed effect, as the perception of depth in the VR environments might influence task performance^109,110^. A subject-specific random effect (i.e., one model intercept parameter per subject) was added to account for intra-subject correlations arising from including both tested body sides for each subject. A Box–Cox transformation was applied on each metric to correct for heteroscedasticity, as subjectively perceived through non-normally distributed model residuals in quantile–quantile plots^111^. Additionally, this transformation allows to capture non-linear effects with the linear models. The models were fitted using maximum-likelihood estimation (MATLAB function *fitlme*) and defined as

1 $$\begin{array}{l}{y}_{i,j}^ {{intact}}={\beta }_{i,0}+{\beta }_{i,1}\ {{\rm{age}}}_{j}+{\beta }_{i,2}\ {{\rm{sex}}}_{j}+{\beta }_{i,3}\ {\text{tested}}\, {\text{body}}\, {\text{side}}_{j}+\\ {\beta }_{i,4}\ {{\rm{handedness}}}_{j}+{\beta }_{i,5}\ {\text{stereo}}\, {\text{vision}}\, {\text{deficits}}_{j}+{W}_{i,j}+{\epsilon }_{i},\end{array}$$

where ${y}_{i,j}^ {{intact}}$ value of a metric *i* of neurologically intact subject *j*

$$\begin{array}{l}{\beta }_{i}\quad \,{\text{model}}\, {\text{parameters}}\,\\ {W}_{i,j}\quad \,{\text{subject}}-{\text{specific}}\, {\text{intercept}}\,\\ {\epsilon }_{i}\quad \,{\text{residual}}\, {\text{error}}.\end{array}$$

For any subject being analyzed, the effect of all confounds on the sensor-based metric was removed based on the fitted models. This generated the value ${\bar{y}}_{i,j}$ of a metric without confounds arising from subject demographics:

2 $$\begin{array}{l}{\bar{y}}_{i,j}={y}_{i,j}-{\beta }_{i,1}\ {\text{age}}_{j}-{\beta }_{i,2}\ {\text{sex}}_{j}-{\beta }_{i,3}\ {\text{tested}}\, {\text{body}}\, {\text{side}}_{j}\\\qquad\;\;-\,{\beta }_{i,4}\ {\text{handedness}}_{j}-{\beta }_{i,5}\ {\text{stereo}}\, {\text{vision}}\, {\text{deficits}}_{j}.\end{array}$$

Furthermore, the corrected values ${\bar{y}}_{i,j}$ were then expressed relative to all neurologically intact subjects (${\bar{y}}_{i}^{{intact}}$) with the goal to standardize the range of all metrics, which simplifies their physiological interpretation and enables the direct comparison of different metrics. Therefore, the normalized value ${\hat{y}}_{i,j}$ was defined relative to the median and variability *d*_*i*_ of all neurologically intact subjects:

3 $${\hat{y}}_{i,j}=\frac{{\bar{y}}_{i,j}-\,{\text{median}}\,\left({\bar{y}}_{i}^{{intact}}\right)}{{d}_{i}},$$

with the median absolute deviation (MAD) of all neurologically intact subjects being used as a variability measure ^112^:

4 $${d}_{i}=\,{\text{median}}\,(\Vert {\bar{y}}_{i,j}^ {{intact}}-\,{\text{median}}\,\left({\bar{y}}_{i}^ {{intact}}\right)\Vert),$$

The MAD was preferred over the standard deviation, as the former allows a more robust analysis that is independent of the underlying distribution of a metric^112^. Lastly, the values ${\hat{y}}_{i,j}$ were divided by the maximal observed value in the included neurological population, such that the subject currently showing worst task-performance receives a score of 100%. In order to discriminate normal from abnormal behavior based on the normalized values, a cut-off was defined based on the 95th percentile (i.e., imposed false positive detection rate of 5%) of each metric ${\hat{y}}_{i}^{{intact}}$ across all neurologically intact subjects.

### Data-driven selection and validation of digital health metrics

The sensor-based metrics were reduced to a subset with optimal clinimetric properties based on three selection steps, followed by two additional validation steps. To evaluate the ability of this selection process to discriminate between physiologically relevant information and random noise, the selection steps were additionally applied to a simulated random metric (simulated Gaussian noise) containing no physiologically relevant information. This metric was constructed by randomly drawing data from a $\mathrm{log}\,$-normal distribution (mean 46.0, standard deviation 32.2, mimicking the distribution of the total time for the reference population) for each subject and tested body side.

### Metric selection and validation: step 1

With the goal to better understand the influence of subject demographics on the sensor-based metric, two-sided simulated likelihood ratio tests (1000 iterations) between the full model and a reduced model without the fixed effect of interest were used to generate *p*-values that were interpreted based on a 5% significance level^113^. This allowed to judge whether a fixed effect influenced the sensor-based metric in a statistically significant manner. We removed metrics that were significantly influenced by stereo vision deficits, as we expected that the influence of stereo vision deficits cannot always be compensated for, for example if their presence is not screened in a clinical setting.

As the performance of the presented confound correction process depends on the fit of the model to the data, we additionally removed metrics with low model quality according to the criteria *C*1 and *C*2, which describe the mean absolute estimation error (MAE) of the models and its variability^114^:

5 $$C{1}_{i}:\frac {{MA{E}}_{i}}{{\rm{range}}\left({y}_{i}^ {{intact}}\right)}\le 15 \%$$

and

6 $$C{2}_{i}:\frac {{MA{E}}_{i}+3\ {\sigma }_{i}}{{\rm{range}}\left({y}_{i}^ {{intact}}\right)}\le 25 \%$$

where $${MAE}=\frac{1}{n}\sum \left\Vert {\epsilon }_{i}^{{intact}}\right\Vert$$

$$\begin{array}{l}n\, = \,{\text{number}}\, {\text{of}}\, {\text{data}}\, {\text{points}}\, {\text{from}}\, {\text{neurologically}}\, {\text{intact}}\, {\text{subjects}}\,\\ {\sigma }_{i}\,=\,{\rm{std}}\left(\left\Vert {\epsilon }_{i}^{{intact}}\right\Vert \right)\\ {std}\,=\,{\text{standard}}\, {\text{deviation}}.\end{array}$$

Fulfilling both criteria leads to the selection of models with moderate and good quality according to the definition of Roy et al.^114^. Before the calculation of *C*1 and *C*2, data points with the 5% highest residuals were removed^114^. The criteria *C*1 and *C*2 were preferred over the more commonly used coefficient of determination *R*^2^, because the magnitude of this metric is highly dependent on the distribution of the dependent variable, which prohibits the definition of a model quality threshold that is valid across metrics^114,115^.

### Metric selection and validation: step 2

ROC analysis was used to judge the potential of a metric to discriminate between neurologically intact and affected subjects, which is a fundamental requirement to validate that the proposed metrics are sensitive to sensorimotor impairments^25,116^. In more detail, a threshold was applied for each metric to classify subjects as being either neurologically intact or impaired. The threshold was varied across the range of all observed values for each metric and the true positive rate (number of subjects correctly classified as neurologically affected divided by the total number of neurologically affected subjects) and false positive rate (number of subjects incorrectly classified as neurologically affected divided by the total number of neurologically intact subjects) were calculated. The area under the curve (AUC) when plotting true positive rates against false positive rates was used as a quality criterion for each metric (Fig. 2).

For metrics to be responsive to intervention-induced physiological changes and allow a meaningful tracking of longitudinal changes, it is fundamental to have low intra-subject variability, high inter-subject variability, and yield repeatable values across a test–retest sessions. Therefore, the data set with 60 neurologically intact subjects performing the VPIT protocol on two separate testing days was used to quantify test–retest reliability. Specifically, the intra-class correlation coefficient (ICC) was calculated to describe the ability of a metric to discriminate between subjects across multiple testing days (i.e., inter-subject variability)^117,118^. The agreement ICC based on a two-way analysis of variance (ICC *A*,*k*) was applied while pooling data across both tested body sides. Further, the smallest real difference (SRD) was used to define a range of values for that the assessment cannot distinguish between measurement error and an actual change in the underlying physiological construct (i.e., intra-subject variability)^119^. For each metric *i*, the SRD was defined as

7 $$SR{D}_{i}=1.96\cdot \sqrt{2}\cdot {\Sigma }_{i}^{intact}\cdot \sqrt{1-IC{C}_{i}}$$

where *Σ*_*i*_ = std across repetitions, subjects, and testing days.

To directly relate the SRD to the distribution of a metric, it was further expressed relative to a metrics’ range:

8 $${\mathrm {SRD}}{ \% }_{i}=100\cdot \frac{\mathrm {{SR{D}}}_{i}}{{\rm{range}}\left({\hat{y}}_{i}^{{intact}}\right)}.$$

Lastly, to distinguish task-related learning from physiological changes when testing subjects before and after receiving an intervention, the presence and strength of learning effects was calculated for each metric. For this purpose, a paired *t*-test was performed between data collected at test and retest to check for a statistically significant difference between the days. Then, the strength (i.e., slope) of the learning effect was estimated by calculating the mean difference between test and retest and normalizing it with respect to the range of observed values:

9 $${\eta }_{i}=100\cdot \frac{{\rm{mean}}({\hat{y}}_{i,j, {retest}}^ {{intact}}-{\hat{y}}_{i,j,test}^ {{intact}})}{{\rm{range}}\left({\hat{y}}_{i}^ {{intact}}\right)}.$$

Metrics passed this second selection step if the AUC did indicate acceptable, excellent, or outstanding discriminant ability (AUC ≥ 0.7) and they had at least acceptable reliability (i.e., ICC values above 0.7)^25,116^. As no cutoff has been defined for the interpretation of the SRD%^120^, we removed the metrics that had the 20% worst SRD% values. Hence, metric passed the evaluation (i.e., small measurement error relative to other metrics) if the SRD% was below 30.3 (80th percentile). Similarly, no cutoff for the interpretation of learning effects was available. Hence, metrics passed the evaluation (i.e., no strong learning effects) if *η* was above −6.35 (20th percentile) of observed values.

### Metric selection and validation: step 3

The correlations between the metrics were analyzed with the goal to identify a set of metrics that contains little redundant information to simplify clinical interpretability. Therefore, a correlation matrix was constructed using partial Spearman correlations. This technique allows to describe the relation between two metrics and to simultaneously model all other metrics that could potentially influence the relationship between the two metrics of interest^121,122^. Hence, this approach can help to exclude certain non-causal correlations. A pair of metrics with an absolute partial correlation *ρ*_p_ of at least 0.5 was considered for removal^123^. From this pair of metrics, the one that had inferior psychometric properties (AUC, ICC, and SRD%) or was less accepted in literature was removed. To simplify the interpretation of the correlation results, we applied the analysis only to metrics that passed all previous selection steps. Additionally, this analysis was applied in an iterative manner, as the removal of certain metrics, which were previously modeled, can change the remaining inter-correlations. The correlation coefficients were interpreted according to Hinkle et al.: very high: *ρ*_p_ ≥ 0.9; high: 0.7 ≤ *ρ*_p_ < 0.9; moderate: 0.5 ≤ *ρ*_p_ < 0.7; low: 0.3 ≤ *ρ*_p_ < 0.5; very low: *ρ*_p_ < 0.3^123^.

### Further validation of metrics: step 1

To better identify the pathophysiological correlates of the metrics that passed all previous evaluation steps, exploratory factor analysis was applied^124–126^. This method tries to associate the variability observed in all metrics with *k* unobserved latent variables via factor loadings, which can be interpreted in light of the initial physiological motivation of the metrics. Exploratory factor analysis was implemented using maximum-likelihood common factor analysis followed by a *promax* rotation (MATLAB function *factoran*). For the interpretation of the emerged latent space, we only considered strong (absolute value ≥ 0.5) factor loadings^124^. The number of factors *k* was estimated in a data-driven manner using parallel analysis (R function *fa.parallel*)^127^. This approach simulates a lower bound that needs to be fulfilled by the eigenvalue associated to each factor and has been shown to be advantageous compared to other more commonly used criteria, such as the Kaiser condition (i.e., eigenvalues >1 are retained)^125,126^. Also, the Kaiser–Meyer–Olkin value (KMO) was calculated to evaluate whether the data was mathematically suitable for the factor analysis.

### Further validation of metrics: step 2

An additional clinically relevant validation step evaluated the ability of the metrics to capture the severity of upper limb disability. For this purpose, each population was grouped according to their disability level as defined by commonly used clinical scores. Subsequently, the behavior of the metrics across the subpopulations and the reference population were statistically analyzed. Stroke subjects were grouped according to the FMA-UE score (ceiling: FMA-UE = 66; mild impairment: 54 ≤ FMA-UE < 66; moderate impairment: 35 ≤ FMA-UE < 54)^128^. MS subjects were split into three groups based on their ARAT score (full capacity: 55 ≤ ARAT ≤ 57; notable capacity: 43 ≤ ARAT < 55; limited capacity: 22 ≤ ARAT < 43)^129^. ARSACS subjects were divided into three different age-groups (young: 26 ≤ age ≤36; mid-age: 37 ≤ age ≤ 47; older-age: 48 ≤ age ≤ 58) due to the neurodegenerative nature of the disease^4^. A Kruskal–Wallis two-sided omnibus test followed by post-hoc tests (MATLAB functions *kruskalwallis* and *multcompare*) were applied to check for statistically significant differences between groups. Bonferroni corrections were applied in both cases.

### Reporting summary

Further information on experimental design is available in the Nature Research Reporting Summary linked to this article.

## Supplementary information

Supplementary Information

Reporting summary checklist

## Data Availability

The datasets used in the current study are available from the corresponding author upon reasonable request and under consideration of the ethical regulations. The haptic end-effector of the VPIT can be purchased at 3D Systems and the force sensors at Pewatron AG.

## Code availability

Source code for the metric selection framework is available at: https://github.com/ChristophKanzler/MetricSelectionFramework. The software for rendering the virtual reality environment and the drawings for the rapid-prototyped handle are available upon reasonable request.
