# Supplementary material for: A data-driven framework for selecting and validating digital health metrics: use-case in neurological sensorimotor impairments
Source: NPJ Digit Med. 2020 May 29;3:80. doi: 10.1038/s41746-020-0286-7 (PMC7260375; doi:10.1038/s41746-020-0286-7)
Supplement: Supplementary file 1 — Supplementary Information [file 41746_2020_286_MOESM1_ESM.pdf]

Supplementary material for:
A data-driven framework for selecting and validating digital health metrics: use-case in neurological sensorimotor impairments

Christoph M. Kanzler, MSc[1], Mike D. Rinderknecht, PhD[1], Anne Schwarz, MSc[2,3],
Ilse Lamers, PhD[4,5], Cynthia Gagnon, PhD[6], Jeremia Held, PhD[2,3], Peter Feys, PhD[4],
Andreas R. Luft, MD[2,3], Roger Gassert, PhD[1],
and Olivier Lambercy, PhD[1]

1 Rehabilitation Engineering Laboratory, Institute of Robotics and Intelligent Systems, Department of Health Sciences and Technology, ETH Zürich, Switzerland.
2 Division of Vascular Neurology and Rehabilitation, Department of Neurology, University Hospital and University of Zürich, Switzerland.
3 cereneo Center for Neurology and Rehabilitation, Vitznau, Switzerland.
4 REVAL, Rehabilitation Research Center, BIOMED, Biomedical Research Institute, Faculty of Medicine and Life Sciences, Hasselt University, Belgium.
5 Rehabilitation and MS center, Pelt, Belgium.
6 School of Rehabilitation, Faculty of Medicine and Health Sciences, Université de Sherbrooke, Québec, Canada.

Corresponding author: Christoph M. Kanzler, Rehabilitation Engineering Laboratory, ETH Zürich, BAA C 307.1, Lengghalde 5, 8008 Zürich, Switzerland.
relab.publications@hest.ethz.ch. +41 44 510 72 34.

Supplementary Analysis

Comparison to existing machine learning algorithms

Methods It is essential to compare the proposed framework to existing machine learning algorithms. Therefore, an extensive comparative analysis using three state of the art machine learning techniques was performed. This included least absolute shrinkage and selection operator (LASSO) regression, random forest-based feature importance weighting, and supersparse linear integer models (SLIM) [1–3].

Starting with the 77 raw VPIT metrics (i.e., without any confound modelling), we applied (a) the LASSO approach, (b) SLIM, and (c) the random forest-based feature weighting to compare the presented results to the established feature selection methods. Subsequently, the metrics chosen by these machine learning techniques were compared to the evaluation with clinimetric properties proposed in this manuscript, as clinimetric properties are the most accepted evidence in neurorehabilitation and other clinical fields.

In more detail, the following variables were normalized as z-scores and used as independent variables for the models: age, sex, tested hand, dominant hand, session (day 1 or day 2), trial (1-5), and the 77 metrics. This implies that data from test and retest session (if available) on a repetition level was used for the analysis, as this is essential to also take the measurement error and test-retestreliability in the data into account. The knowledge about the history of neurological injury (binary) was used as dependent variable.

For the LASSO, a leave-one-out cross-validation was performed to choose the optimal value of the parameter Lambda (Supplementary Figure 1). The trade-off parameter C of SLIM was defined as 0.005. The random forest was trained using 100 trees and permutation importance was used as statistical criteria to characterize the relevance of the features.

For this particular subanalysis, we hypothesized that each methodology will identify a different core set that is optimal according to the respective optimization criteria.

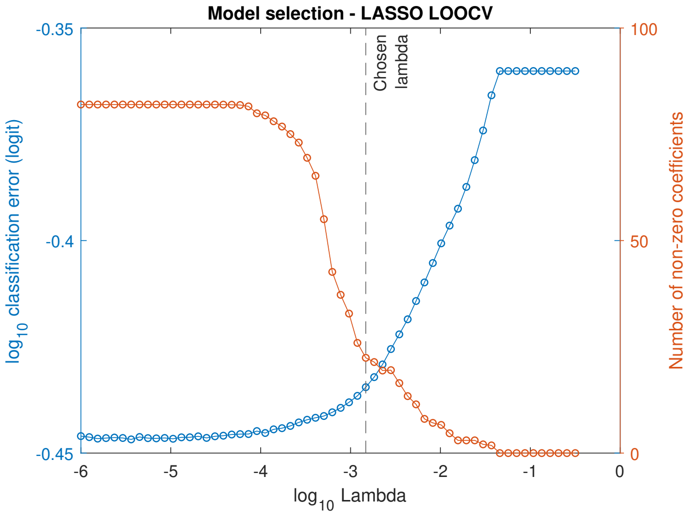

Supplementary Figure 1: **Selection of parameter lambda for the LASSO.** By increasing Lambda, the model tries to reduce the number of used features (i.e. decreases the number of non-zero model coefficients). The model performs best when relying on all the features, and the loss increases as the number of used features decreases (with larger lambda). We want to select a subset of features that can still be used to accurately discriminate between neurologically intact and affected individuals. Hence, we need to have a trade-off between classification error and number of features. Lambda was chosen as the value before the exponen-tial increase of the error. The loss increases slowly up to that point, meaning that the discarded metrics had little effect on the correctness of the classification, and were discarded during model training.

Results The results for the LASSO, SLIM, and random forest in comparison to the clinimetric properties can be found in Table 1, 2, and 3 respectively.

The LASSO approach selected 16 VPIT metrics in the final core set. These metrics did fulfil most, but not all of the proposed criteria for the clinimetric properties, and only two of them were also part of the core set proposed in the manuscript. The AUC cut-off of 0.7 was fulfilled by 7

Supplementary Table 1: **Results for the metric selection using LASSO and comparison with their clinimetric properties.** The metrics in bold highlight the ones that were also selected by the proposed framework as these fullfilled the evaluation criteria of all clinimetric properties. SPARC: spectral arc length. GF: grip force. TP: transport. RT: return. HA: hole approach.

Selected metrics	AUC	ICC	SRD%	Learning rate	Confounds modeling
Age	-	-	-	-	-
Gender	-	-	-	-	-
Tested hand	-	-	-	-	-
Trial	-	-	-	-	-
Velocity max TP	0.83	0.87	18.57	-9.14	Sufficient
Haptic collisions max TP	0.63	0.84	20.54	-1.08	Sufficient
Haptic collisions mean RT	0.61	0.72	25.32	-0.07	Sufficient
Haptic collisions max RT	0.46	0.79	27.02	4.37	Insufficient
SPARC TP	0.84	0.83	23.78	-7.16	Insufficient
Time to max velocity TP	0.45	0.78	28.70	3.93	Insufficient
Throughput TP	0.92	0.81	24.07	-12.18	Insufficient
Throughput RT	0.90	0.78	27.43	-13.21	Sufficient
GF rate SPARC RT	0.64	0.78	23.81	-6.35	Sufficient
**Log jerk RT**	0.73	0.75	25.33	-6.08	Sufficient
Trajectory error mean RT	0.56	0.84	20.00	1.24	Sufficient
Initial mov angle RT θ_1	0.51	0.75	33.90	3.18	Sufficient
Log jerk hole approach	0.57	0.68	30.60	-4.84	Sufficient
GF mean RT	0.49	0.76	27.62	0.17	Sufficient
Force buildup time	0.70	0.82	21.36	-6.97	Sufficient
**FR SPARC TP**	0.74	0.82	22.48	-5.71	Sufficient

Supplementary Table 2: **Results for the metric selection using SLIM and comparison with their clinimetric properties.** The metrics in bold highlight the ones that were also selected by the proposed framework as these fullfilled the evaluation criteria of all clinimetric properties. SPARC: spectral arc length. GF: grip force. TP: transport. RT: return. HA: hole approach.

Selected metrics	AUC	ICC	SRD%	Learning rate	Confounds modeling
SPARC TP	0.84	0.83	23.78	-7.16	Insufficient
**Log jerk TP**	0.78	0.74	26.11	-4.82	Sufficient
Haptic collisions max TP	0.61	0.84	24.55	-3.99	Sufficient
Number velocity peaks TP	0.82	0.79	21.30	-6.36	Insufficient
Tested hand	-	-	-	-	-
**GF rate SPARC HA**	0.84	0.82	26.38	-5.94	Sufficient
GF rate num. peaks HA	0.84	0.82	26.38	-5.94	Sufficient
Gender	-	-	-	-	-
GF num peaks buildup	0.15	0.44	57.70	0.77	Insufficient
GF mean RT	0.49	0.76	27.62	0.17	Sufficient

Supplementary Table 3: **Results for the metric selection using the random forest and comparison with their clinimetric properties.** The 10 variables that yielded the highest feature importance ranking according a random forest are visualized. The metrics in bold highlight the ones that were also selected by the proposed framework as these fullfilled the evaluation criteria of all clinimetric properties.

Selected metrics	AUC	ICC	SRD%	Learning rate	Confounds modeling quality
Session	-	-	-	-	-
Endpoint error TP	-	-	-	-	-
Age	-	-	-	-	-
GF rate mean RT	0.07	0.82	27.79	5.87	Sufficient
Velocity max TP	0.83	0.87	18.57	-9.14	Sufficient
GF rate max RT	0.29	0.48	34.05	7.19	Sufficient
Throughput TP	0.92	0.81	24.07	-12.18	Insufficient
Velocity mean TP	0.83	0.88	20.61	-9.99	Sufficient
**Velocity max RT**	0.83	0.87	18.57	-6.27	Sufficient
GF buildup duration	0.70	0.82	21.36	-6.97	Sufficient

metrics and 4 of the remaining ones had AUCs of at least 0.6. Five metrics had an AUC smaller than 0.6. All metrics except log jerk hole approach fulfilled the ICC and SRD criteria. The learning rate criteria was not fulfilled by five metrics. For five of the selected metrics, the mixed effects models could not accurately compensate for learning effects. The partial inter-metric correlations of the selected feature sets were all below 0.5 except for throughput transport (TP) and log jerk hole approach (HA), as well as initial movement angle return (RT) θ_1 and trajectory error mean RT.

SLIM selected eight VPIT metrics in the final core set. These metrics did fulfil most, but not all of criteria established for the clinimetric properties, and only two of them were also part of the core set proposed in the manuscript. In more detail, the AUC cut-off of 0.7 was fulfilled by five metrics. The ICC criteria was fulfilled by all selected metrics expect one. The learning rate criteria was fulfilled by si metrics. For three metrics, mixed effect models could not accurately compensate for confounds. The partial inter-correlations of the selected core set were below 0.5, except for grip force (GF) rate SPARC HA and GF rate peaks HA.

The best VPIT metrics according to the random forest did fulfil most, but not all criteria of the clinimetric properties, and only two of the metrics were also part of the core set proposed in the manuscript. Seven metrics showed AUCs above 0.7, and the remaining three had AUCs of 0.07, 0.29, and 0.49. All metrics expect GF rate max RT fulfilled the evaluation criteria for the ICC and SRD. Four of the metrics did not show strong learning effects. For two of the selected metrics, the mixed effect models could not accurately compensate for confounds. Four combinations of the metrics showed partial inert-correlations above 0.5 for the random forest.

Discussion As expected, each of the feature selection methods (LASSO, SLIM, random forest, and the proposed clinimetrics-based selection framework) led to a different core set of selected final metrics. This reflects the fact that these core sets are optimal according to the defined mathematical loss function of each method. The metrics selected by LASSO, SLIM, and random forest did fulfil most, but not all, of the clinimetric properties. The models selected metrics that have a low

AUC value (e.g., GF rate mean RT of 0.07). This is likely because this metric itself does not allow to accurately discriminate healthy and neurologically affected subjects, but, in combination with all other metrics, it helps the model to discriminate the two classes (see Figure 1 in [4]). However, this is not necessarily a desirable property for a metric when attempting to evaluate the effect of an intervention on sensorimotor impairments, as each metric on its own is expected to carry insightful information about the impairments. Interestingly, the metrics selected by LASSO, SLIM, and random forest fulfilled almost all of the ICC and SRD evaluation criteria. Hence, it seems that metrics with high intra-subject variability, low inter-subject variability, and low repeatability across sessions are automatically filtered out by the models. Metrics with strong learning effects were not filtered by the LASSO and random forests, even though SLIM performed decently (only two metrics with strong learning effects). Lastly, multiple metrics were included in the core sets by LASSO, SLIM, and random forest, even though the mixed effect models were not able to accurately compensate for confounds for these metrics. In these cases, it is unclear if the LASSO, SLIM, and random forest were able to accurately compensate for confounds or not.

While the metrics selected by the LASSO and SLIM had mostly very low inter-correlation, the ones selected by the random forest were partly redundant. This is in line with optimization criteria of these selection methods (i.e., LASSO and SLIM minimize inter-correlations, whereas a random forest does not explicitly take them into account).

Conclusions All in all, these analyses suggest that the stepwise clinimetric-based selection provides a conservative and transparent method that is well-suited for selecting metrics in the specific application of repeatedly assessing impairments. This makes it an interesting approach for researchers in the field of digital health and an alternative to more established feature selection algorithms, which are not optimized to consider all relevant clinimetric properties of a metric. However, the proposed method is not as flexible as the LASSO and random forest, which can be implemented in the context of many other applications and always yield a mathematically optimal solution.

Supplementary Methods

Participants

Neurologically intact subjects were recruited at ETH Zurich (Zurich, Switzerland). Stroke patients were tested at the University Hospital of Zurich (Zurich, Switzerland), the cereneo Center for Neurology and Rehabilitation (Vitznau, Switzerland), and the Zentrum für ambulante Rehabilitation (ZAR, Zurich, Switzerland) as part of the Study of Motor Learning and Acute Recovery Time Course in Stroke (SMARTS) or the synergy-based open-source foundations and technologies for prosthetics and rehabilitation (SoftPro). Multiple sclerosis (MS) patients were recruited at Hasselt University (Hasselt, Belgium), at KU Leuven (Leuven, Belgium), and at the Rehabilitation and MS Center Pelt (Pelt, Belgium). Autosomal-recessive-spastic-ataxia of Charlevoix-Saguenay (ARSACS) patients were included at the Neuromuscular Clinic of the Centre de Sante et de Services Sociaux de Jonquière (Jonquière, Canada). Exclusion criteria involved the inability to lift the arm against gravity, to flex/extend the fingers, and the presence of any concomitant disease affecting the upper limb. The studies involving stroke patients additionally used increased muscle tone, severe sensory deficits, hemorrhagic infarct, traumatic brain injury as exclusion criteria. MS patients had to be diagnosed according to the McDonald criteria. All clinical assessments were performed within the same or few days of the Virtual Peg Insertion Test (VPIT) assessment.

Data preprocessing

First, temporal gaps larger than 50 samples in the recorded position, force, and haptic time-series were linearly interpolated. Such gaps can stem from a delayed communication between the soft- and hardware components during the data recordings. Subsequently, a 1D trajectory $d(t)$ defining the distance d covered until timepoint t was estimated from the 3D cartesian position trajectories p_x, p_y, and p_z by summing up their absolute first time-derivatives relative to the start of the repetition:

$$d(t) = \sum_{1}^{t} \|\dot{p}_x\| + \|\dot{p}_y\| + \|\dot{p}_z\| \tag{1}$$

Afterwards, velocity (first time-derivative) and jerk (third time-derivative) signals were derived from $d(t)$. Also, single grasping force and grip force rate (first time-derivative) trajectories were generated by averaging across the signals of the three piezoresistive sensors. All time-series were low-pass filtered initially and after each derivation using a zero-phase Butterworth filter (4th order, cut-off frequency 8 Hz). Data from an entire peg were removed if it was dropped and not inserted into a hole before another peg was picked up, which occurred for 1.6% of all pegs.

To isolate rapid ballistic movements, the trajectories of each peg were segmented into the *transport* (i.e., ballistic movement while transporting the peg to a hole) and *return* (i.e., ballistic movement while returning the cursor to the next peg) phases (Supplementary Figure 1). The *transport* phase started at the last occasion the velocity exceeded a threshold $\theta_{vel,tp}$ after the peg was picked up and before maximum velocity $v_{max,tp}$ was reached. The threshold $\theta_{vel,tp}$ was set to 10% of $v_{max,tp}$ that occurred before the insertion of the peg into the next hole. The end of the *transport* was defined as the first time the velocity dropped below $\theta_{vel,tp}$ after $v_{max,tp}$. To ensure a robust segmentation, the *transport* phase of a peg was discarded in case the peg was taken at $v_{max,tp}$, the velocity never dropped below $\theta_{vel,tp}$ after $v_{max,tp}$ before oreleasing the peg,r the length

of the phase was below 0.1 s. The same criteria were applied to segment the *return* phase, which was defined as the main ballistic movement component between releasing a peg and picking up the next peg, given the maximal velocity $v_{max,rt}$ during return and $\theta_{vel,rt}$. For segmenting the *transport* and *return* phases, only the horizontal component of $d(t)$ was used. For the analyzed pegs, approximately 0.43% of the transport, 2.5% of the return, 2.89% of the peg approach, and 0.31% hole approach phases were removed in neurologically affected subjects due to the velocity criteria.

To isolate the overshoot when reaching for a target as well as the precise position adjustments related to virtual object manipulations, the trajectories were additionally segmented into the *peg approach* and *hole approach* phases. The former was defined from the end of the *return* until the next peg was picked up. The latter was defined from the end of the *transport* until the current peg was inserted into a hole.

Further, grasping forces were additionally segmented into the *force buildup* (i.e., behaviour during the most rapid production of force) and *force release* phases (i.e., behaviour during the most rapid release of force), by first identifying the position of the maximum and minimum value in grip force rate between approaching and inserting each peg (Supplementary Figure 1). Subsequently, the start and end of the *force buildup* phase was defined as the last and first time the grip force rate was below 10% of its maximum before and after the maximum, respectively. Similarly, the start and end of the *force release* phase was determined based on the last and first time the grip force rate was above 10% of its minimum value before and after the minimum, respectively.

Supplementary Results

The metrics that did not fullfil the required quality of the models, according to the C1 and C2 criteria, were *spectral arc length transport*, *number of velocity peaks transport*, *distance to max. velocity transport*, *time to max. velocity transport*, *number of velocity peaks return*, *throughput transport*, *initial movement angle transport* θ_1, *initial movement angle* θ_2, *collision force max. return*, *grip force rate number of peaks buildup*, *grip force rate spectral arc length buildup*, *grip force rate number of peaks release*, and *simulated Gaussian noise*. The metrics that were altered by stereo vision deficits were *initial movement angle transport* θ_1, θ_2, θ_3, *number of movement ends*, *number of dropped pegs*, and *grip force rate number of peaks buildup*.

Supplementary Table 4: **Detailed demographics and clinical information for each body side of each included neurologically impaired subject.**

**Disease**	**Age** (yrs)	**Sex**	**Tested side**	**Affected side**	**Dominant side**	**Chronicity** (yrs)	**FMA-UE** (0-66)	**ARAT** (0-57)	**NHPT** (s)	**EDSS** (0-10)
Stroke	67	Male	Right	Left	Right	2.09	66	57	23.25	-
Stroke	55	Male	Left	Left	Right	1.69	54	56	33.25	-
Stroke	55	Male	Right	Left	Right	1.69	66	57	21.85	-
Stroke	55	Male	Left	Right	Right	2.01	65	57	22.82	-
Stroke	55	Male	Right	Right	Right	2.01	49	55	29.28	-
Stroke	52	Male	Left	Left	Right	2.74	55	52	35.36	-
Stroke	52	Male	Right	Left	Right	2.74	65	57	20.99	-
Stroke	73	Male	Left	Right	Right	0.89	62	-	-	-
Stroke	69	Female	Right	Left	Right	0.86	61	57	20.32	-
Stroke	67	Male	Left	Left	Right	2.42	50	-	-	-
Stroke	67	Male	Right	Left	Right	2.42	66	-	-	-
Stroke	40	Female	Left	Right	Right	0.77	56	45	-	-
Stroke	40	Female	Right	Right	Right	0.77	49	49	-	-
Stroke	71	Male	Left	Left	Left	4.49	40	35	196.69	-
Stroke	71	Male	Right	Left	Left	4.49	65	57	15.03	-
Stroke	59	Female	Left	Left	Right	4.35	50	47	17.70	-
Stroke	59	Female	Right	Left	Right	4.35	66	57	12.57	-
Stroke	88	Female	Left	Left	Right	1.65	37	39	42.17	-
Stroke	88	Female	Right	Left	Right	1.65	63	-	14.33	-
Stroke	69	Female	Left	Right	Right	0.58	63	57	19.81	-
Stroke	69	Female	Right	Right	Right	0.58	44	39	49.16	-
Stroke	59	Female	Left	Right	Right	1.94	66	57	21.50	-
Stroke	59	Female	Right	Right	Right	1.94	57	56	21.63	-
Stroke	50	Female	Right	Left	Right	4.83	64	-	-	-
Stroke	61	Male	Left	Right	Right	8.70	66	56	24.51	-
Stroke	61	Male	Right	Right	Right	8.70	38	42	34.95	-
Stroke	59	Male	Left	Left	Right	1.64	46	40	40.84	-
Stroke	59	Male	Right	Left	Right	1.64	63	57	14.85	-
Stroke	69	Male	Left	Left	Right	0.51	53	51	23.08	-
Stroke	69	Male	Right	Left	Right	0.51	63	56	13.67	-
Stroke	55	Male	Left	Left	Right	1.45	59	57	28.08	-
Stroke	55	Male	Right	Left	Right	1.45	66	57	18.50	-
Stroke	42	Male	Left	Left	Right	0.48	39	30	-	-
Stroke	42	Male	Right	Left	Right	0.48	65	57	20.47	-
Stroke	51	Female	Left	Right	Right	0.97	66	57	21.01	-
Stroke	51	Female	Right	Right	Right	0.97	61	57	25.70	-
Stroke	58	Male	Left	Right	Right	0.48	62	57	23.33	-
Stroke	58	Male	Right	Right	Right	0.48	42	53	26.00	-
Stroke	46	Male	Left	Left	Right	1.05	57	42	24.03	-
Stroke	46	Male	Right	Left	Right	1.05	66	57	23.09	-
Stroke	76	Male	Left	Right	Right	2.74	66	55	39.73	-
Stroke	76	Male	Right	Right	Right	2.74	60	54	29.19	-

Supplementary Table 4: **Continued.**

**Disease**	**Age** (yrs)	**Sex**	**Tested side**	**Affected side**	**Dominant side**	**Chronicity** (yrs)	**FMA-UE** (0-66)	**ARAT** (0-57)	**NHPT** (s)	**EDSS** (0-10)
Stroke	53	Female	Left	Right	Right	2.98	66	57	22.99	-
Stroke	53	Female	Right	Right	Right	2.98	58	55	20.67	-
Stroke	62	Male	Left	Right	Right	14.65	66	57	19.58	-
Stroke	62	Male	Right	Right	Right	14.65	34	33	154.00	-
Stroke	62	Male	Left	Right	-	-	-	57	24.60	-
Stroke	62	Male	Right	Right	-	-	-	43	86.00	-
Stroke	54	Female	Left	Left	Right	1.00	66	57	-	-
Stroke	54	Female	Right	Left	Right	1.00	66	57	-	-
Stroke	67	Male	Left	Left	Right	0.46	66	57	-	-
Stroke	67	Male	Right	Left	Right	0.46	66	57	-	-
Stroke	52	Male	Left	Left	Right	0.23	66	57	-	-
Stroke	52	Male	Right	Left	Right	0.23	66	57	-	-
Stroke	46	Male	Left	Right	Right	0.23	66	57	-	-
Stroke	71	Male	Left	Left	Right	0.23	64	57	-	-
Stroke	71	Male	Right	Left	Right	0.23	66	57	-	-
Stroke	48	Male	Left	Right	Right	0.02	57	57	-	-
Stroke	48	Male	Right	Right	Right	0.02	66	47	-	-
Stroke	45	Female	Right	Left	Right	0.02	66	57	-	-
Stroke	55	Female	Right	Left	Right	0.08	66	57	-	-
Stroke	65	Male	Left	Left	Right	0.23	60	-	-	-
Stroke	65	Male	Right	Left	Right	0.02	62	53	-	-
Stroke	43	Male	Left	Right	Right	0.46	66	57	-	-
Stroke	43	Male	Right	Right	Right	0.46	66	56	-	-
Stroke	41	Female	Right	Left	Right	0.02	64	-	-	-
Stroke	35	Male	Left	Left	Right	0.46	61	57	-	-
Stroke	35	Male	Right	Left	Right	0.02	64	57	-	-
Stroke	76	Male	Right	Left	Left	0.23	66	57	-	-
Stroke	86	Male	Right	Left	Right	0.02	62	56	-	-
Stroke	50	Male	Left	Left	Left	1.00	65	57	-	-
Stroke	49	Male	Right	Left	Left	0.23	66	57	-	-
Stroke	74	Male	Left	Right	Right	0.02	66	57	-	-
Stroke	81	Female	Left	Right	Right	0.23	66	57	-	-
Stroke	65	Female	Left	Left	Right	0.23	66	56	-	-
Stroke	65	Female	Right	Left	Right	0.23	66	57	-	-
Stroke	21	Male	Left	Right	Right	0.02	63	57	-	-
Stroke	21	Male	Right	Right	Right	0.02	66	56	-	-
Stroke	87	Female	Left	Right	Left	0.02	66	57	-	-
Stroke	87	Female	Right	Right	Left	0.02	50	29	-	-
Stroke	54	Male	Left	Right	Left	0.46	66	57	-	-
Stroke	54	Male	Right	Right	Left	0.46	54	57	-	-
Stroke	57	Male	Left	Left	Right	0.02	66	57	-	-
Stroke	57	Male	Right	Left	Right	0.02	61	57	-	-
Stroke	70	Female	Right	Left	Right	0.53	66	-	16.46	-

Supplementary Table 4: **Continued.**

**Disease**	**Age** (yrs)	**Sex**	**Tested side**	**Affected side**	**Dominant side**	**Chronicity** (yrs)	**FMA-UE** (0-66)	**ARAT** (0-57)	**NHPT** (s)	**EDSS** (0-10)
Stroke	57	Male	Right	Right	-	0.48	66	-	22.61	-
Stroke	73	Male	Left	Left	Right	0.53	63	-	28.55	-
Stroke	56	Male	Left	Left	Right	0.03	25	-	60.81	-
Stroke	63	Male	Left	Right	Right	0.48	66	-	14.33	-
ARSACS	41	Female	Left	Both	Right	-	-	-	28.59	-
ARSACS	41	Female	Right	Both	Right	-	-	-	37.14	-
ARSACS	29	Male	Left	Both	Right	-	-	-	56.98	-
ARSACS	29	Male	Right	Both	Right	-	-	-	40.34	-
ARSACS	56	Female	Left	Both	Left	-	-	-	83.59	-
ARSACS	56	Female	Right	Both	Left	-	-	-	95.20	-
ARSACS	37	Male	Left	Both	Left	-	-	-	36.36	-
ARSACS	37	Male	Right	Both	Left	-	-	-	46.72	-
ARSACS	26	Female	Left	Both	Right	-	-	-	-	-
ARSACS	26	Female	Right	Both	Right	-	-	-	-	-
ARSACS	37	Female	Left	Both	Right	-	-	-	-	-
ARSACS	37	Female	Right	Both	Right	-	-	-	-	-
ARSACS	31	Male	Left	Both	Right	-	-	-	29.88	-
ARSACS	31	Male	Right	Both	Right	-	-	-	23.52	-
ARSACS	58	Male	Left	Both	Right	-	-	-	60.43	-
ARSACS	58	Male	Right	Both	Right	-	-	-	47.33	-
MS	52	Female	Left	Both	Right	29.00	-	37	45.25	7.0
MS	52	Female	Right	Both	Right	29.00	-	47	24.75	7.0
MS	69	Male	Right	Both	Right	19.00	-	44	140.27	7.5
MS	25	Female	Left	Both	Right	6.00	-	52	29.35	6.0
MS	25	Female	Right	Both	Right	6.00	-	53	29.62	6.0
MS	42	Female	Left	Both	Right	1.00	-	56	27.81	4.0
MS	42	Female	Right	Both	Right	1.00	-	54	20.48	4.0
MS	59	Female	Left	Both	Left	5.00	-	49	27.76	7.0
MS	56	Female	Left	Both	Right	10.00	-	49	33.72	7.0
MS	56	Female	Right	Both	Right	10.00	-	29	89.79	7.0
MS	65	Male	Left	Both	Left	19.00	-	52	39.90	8.0
MS	63	Female	Left	Both	Right	8.00	-	57	20.84	4.5
MS	63	Female	Right	Both	Right	8.00	-	54	35.04	4.5
MS	76	Female	Left	Both	Right	38.00	-	43	27.01	5.0
MS	76	Female	Right	Both	Right	38.00	-	34	34.46	5.0
MS	60	Male	Left	Both	Right	21.00	-	52	31.48	7.0
MS	60	Male	Right	Both	Right	21.00	-	53	25.29	7.0
MS	42	Female	Right	Both	Right	21.00	-	39	74.39	7.5
MS	46	Male	Left	Both	Right	11.00	-	55	30.58	5.5
MS	46	Male	Right	Both	Right	11.00	-	56	23.23	5.5
MS	70	Female	Left	Both	Right	37.00	-	53	29.86	6.0
MS	70	Female	Right	Both	Right	37.00	-	45	53.21	6.0
MS	36	Female	Right	Both	Right	6.76	61	56	22.87	7.5

Supplementary Table 4: **Continued.**

**Disease**	**Age** (yrs)	**Sex**	**Tested side**	**Affected side**	**Dominant side**	**Chronicity** (yrs)	**FMA-UE** (0-66)	**ARAT** (0-57)	**NHPT** (s)	**EDSS** (0-10)
MS	40	Male	Right	Both	Left	12.55	53	44	56.17	7.5
MS	35	Male	Left	Both	Right	0.97	65	57	22.90	4.5
MS	35	Male	Right	Both	Right	0.97	65	52	24.90	4.5
MS	52	Female	Left	Both	Right	9.66	62	56	35.13	5.5
MS	52	Female	Right	Both	Right	9.66	65	56	29.31	5.5
MS	65	Female	Right	Both	Both	14.48	61	52	55.37	7.5
MS	53	Male	Right	Both	Right	9.66	62	56	23.93	2.5
MS	59	Male	Left	Both	Right	1.93	63	56	28.70	4.0
MS	59	Male	Right	Both	Right	1.93	63	55	47.49	4.0
MS	35	Female	Left	Both	Right	14.48	62	51	50.17	7.5
MS	38	Male	Left	Both	Right	2.90	-	-	-	3.5
MS	38	Male	Right	Both	Right	2.90	-	-	-	3.5
MS	66	Female	Left	Both	Left	16.42	-	-	-	7.5
MS	66	Female	Right	Both	Left	16.42	-	-	-	7.5
MS	22	Male	Left	Both	Right	3.86	-	-	-	6.5
MS	22	Male	Right	Both	Right	3.86	-	-	-	6.5
MS	38	Female	Left	Both	Right	8.69	-	-	-	7.0
MS	38	Female	Right	Both	Right	8.69	-	-	-	7.0
MS	61	Male	Left	Both	Right	5.79	-	-	20.00	5.0
MS	61	Male	Right	Both	Right	5.79	-	-	26.27	5.0
MS	63	Female	Right	Both	Right	6.76	-	-	28.00	6.0
MS	63	Male	Right	Both	Right	29.93	-	-	16.00	3.0

Supplementary Table 5: **Influence of potential confounds on each sensor-based metric**. For each metric, a mixed effect model was fitted to the Box-Cox-transformed outcome measure. Bold entries indicate that the fixed effect contributed in a statistically significant manner to model quality according to a simulated likelihood ratio test or that model quality was at least moderate according to the criteria $C1$ and $C2$. Abbreviations: SE: standard error; SD: standard deviation. R^2: adjusted coefficient of determination. SPARC: spectral arc length.

Sensor-based metric	Fixed effects												Subject-specific effects	R^2	Model quality
	(Intercept)		Age		Sex		Tested side		Hand dominance		Stereo vision		(Intercept)		
	Estimate (SE)	p-value	Estimate (SE)	p-value	Estimate (SE)	p-value	Estimate (SE)	p-value	Estimate (SE)	p-value	Estimate (SE)	p-value	SD		C1 (%), C2 (%)
Jerk transport	**-30.59 (2.50)**	**0.001**	**0.08 (0.03)**	**0.007**	-0.47 (0.91)	0.604	0.41 (1.09)	0.691	0.78 (1.09)	0.5	-0.59 (1.57)	0.718	3.21	0.20	**5.87, 19.07**
Log jerk transport	**0.27 (0.00)**	**0.001**	**0.00 (0.00)**	**0.005**	0.00 (0.00)	0.734	0.00 (0.00)	0.793	0.00 (0.00)	0.519	0.00 (0.00)	0.879	0.00	0.43	**6.68, 20.55**
Spectral arc length transport	**0.16 (0.00)**	**0.001**	**0.00 (0.00)**	**0.001**	0.00 (0.00)	0.929	**0.00 (0.00)**	**0.027**	0.00 (0.00)	0.995	0.00 (0.00)	0.467	0.00	0.40	8.32, 27.27
Number of velocity peaks transport	-0.05 (0.07)	0.444	**0.00 (0.00)**	**0.001**	0.00 (0.02)	0.872	-0.05 (0.03)	0.142	0.01 (0.03)	0.783	-0.02 (0.04)	0.655	0.07	0.30	8.48, 34.79
Distance to max. velocity transport	**13.77 (0.41)**	**0.001**	0.00 (0.00)	0.613	0.02 (0.14)	0.884	-0.41 (0.21)	0.055	-0.08 (0.21)	0.724	-0.04 (0.25)	0.873	0.23	0.01	9.88, 29.99
Time to max. velocity transport	**14.83 (0.58)**	**0.001**	0.00 (0.01)	0.877	-0.09 (0.21)	0.677	-0.24 (0.28)	0.410	0.08 (0.28)	0.775	-0.18 (0.35)	0.607	0.54	0.03	10.54, 31.32
Jerk return	**-9.88 (0.69)**	**0.001**	**0.03 (0.01)**	**0.001**	-0.32 (0.26)	0.220	-0.26 (0.28)	0.346	0.10 (0.28)	0.724	-0.29 (0.44)	0.505	1.00	0.36	**7.11, 24.36**
Log jerk return	**0.24 (0.00)**	**0.001**	**0.00 (0.00)**	**0.002**	0.00 (0.00)	0.4	0.00 (0.00)	0.293	0.00 (0.00)	0.751	0.00 (0.00)	0.690	0.00	0.49	**7.23, 22.71**
Spectral arc length return	**0.25 (0.01)**	**0.001**	**0.00 (0.00)**	**0.028**	0.00 (0.00)	0.077	0.00 (0.00)	0.096	0.00 (0.00)	0.887	0.00 (0.00)	0.418	0.01	0.42	**6.29, 18.82**
Number of velocity peaks return	0.12 (0.09)	0.189	**0.00 (0.00)**	**0.003**	-0.06 (0.03)	0.065	0.03 (0.04)	0.455	0.04 (0.04)	0.374	-0.07 (0.05)	0.255	0.10	0.17	11.52, 36.43
Distance to max. velocity return	**632.23 (63.79)**	**0.001**	-0.61 (0.68)	0.380	-6.84 (22.91)	0.780	41.14 (29.75)	0.178	25.56 (29.75)	0.395	56.23 (39.31)	0.167	69.24	0.08	**7.22, 22.56**
Time to max. velocity return	**86.45 (7.70)**	**0.001**	-0.04 (0.08)	0.622	1.09 (2.79)	0.727	4.35 (3.47)	0.213	-0.03 (3.47)	0.992	6.21 (4.78)	0.198	9.15	0.12	**7.76, 24.86**
Path length ratio transport	**-3.11 (0.32)**	**0.001**	**0.02 (0.00)**	**0.001**	**0.32 (0.12)**	**0.009**	**0.35 (0.13)**	**0.009**	-0.09 (0.13)	0.5	-0.11 (0.20)	0.585	0.46	0.47	**3.01, 11.43**
Throughput transport	**2.36 (0.18)**	**0.001**	**-0.02 (0.00)**	**0.001**	**-0.36 (0.07)**	**0.001**	-0.03 (0.08)	0.690	0.02 (0.08)	0.805	0.04 (0.12)	0.729	0.24	0.65	8.27, 25.53
Path length ratio return	**-2.80 (0.29)**	**0.001**	**0.02 (0.00)**	**0.001**	**0.40 (0.11)**	**0.004**	**0.59 (0.11)**	**0.001**	-0.08 (0.11)	0.479	0.19 (0.19)	0.317	0.46	0.60	**2.95, 11.10**
Throughput return	**2.59 (0.18)**	**0.001**	**-0.03 (0.00)**	**0.001**	**-0.38 (0.07)**	**0.001**	**-0.36 (0.08)**	**0.001**	0.03 (0.08)	0.701	-0.17 (0.12)	0.133	0.23	0.69	**7.77, 22.93**
Trajectory error mean transport	**0.25 (0.00)**	**0.001**	0.00 (0.00)	0.930	0.00 (0.00)	0.332	**0.00 (0.00)**	**0.001**	0.00 (0.00)	0.976	0.00 (0.00)	0.896	0.00	0.54	**7.61, 22.75**
Trajectory error max transport	**0.25 (0.00)**	**0.001**	0.00 (0.00)	0.269	0.00 (0.00)	0.208	**0.00 (0.00)**	**0.001**	0.00 (0.00)	0.933	0.00 (0.00)	0.280	0.00	0.55	**7.35, 21.98**
Initial movement angle transport θ_1	-0.01 (1.99)	0.998	0.04 (0.02)	0.059	-1.28 (0.72)	0.105	**5.41 (0.90)**	**0.001**	-0.15 (0.90)	0.876	**3.65 (1.24)**	**0.006**	2.34	0.39	8.25, 25.74
Initial movement angle transport θ_2	0.08 (2.03)	0.963	0.04 (0.02)	0.093	-1.33 (0.73)	0.082	**5.42 (0.92)**	**0.001**	-0.23 (0.92)	0.812	**3.74 (1.26)**	**0.004**	2.35	0.39	8.29, 25.89
Initial movement angle transport θ_3	-0.63 (1.45)	0.675	0.03 (0.02)	0.097	-0.77 (0.53)	0.165	**4.59 (0.62)**	**0.001**	0.34 (0.62)	0.584	**1.97 (0.91)**	**0.044**	1.89	0.46	**7.48, 23.81**
Trajectory error mean return	**0.25 (0.00)**	**0.001**	0.00 (0.00)	0.3	0.00 (0.00)	0.191	**0.00 (0.00)**	**0.003**	0.00 (0.00)	0.431	0.00 (0.00)	0.168	0.00	0.40	**4.55, 14.40**
Trajectory error max return	**0.25 (0.00)**	**0.001**	0.00 (0.00)	0.432	0.00 (0.00)	0.385	**0.00 (0.00)**	**0.006**	0.00 (0.00)	0.360	0.00 (0.00)	0.333	0.00	0.39	**5.68, 17.36**
Initial movement angle return θ_1	**14.38 (3.10)**	**0.001**	-0.05 (0.03)	0.170	1.00 (1.12)	0.382	-2.25 (1.43)	0.126	-0.30 (1.43)	0.843	1.17 (1.92)	0.563	3.51	0.11	**5.59, 18.87**
Initial movement angle return θ_2	**14.00 (3.38)**	**0.001**	-0.05 (0.04)	0.188	1.24 (1.22)	0.284	-2.13 (1.56)	0.187	-0.23 (1.56)	0.891	1.27 (2.09)	0.555	3.75	0.10	**5.58, 18.76**
Initial movement angle return θ_3	**10.12 (1.99)**	**0.001**	-0.04 (0.02)	0.071	0.74 (0.72)	0.324	**-2.49 (0.89)**	**0.012**	0.00 (0.89)	1	0.88 (1.24)	0.473	2.41	0.20	**3.89, 12.19**
Velocity mean transport	**19.94 (1.15)**	**0.001**	**-0.09 (0.01)**	**0.001**	**-1.06 (0.44)**	**0.017**	**0.94 (0.34)**	**0.006**	-0.34 (0.34)	0.304	1.05 (0.75)	0.184	2.09	0.79	**6.07, 20.78**
Velocity max transport	**22.05 (1.10)**	**0.001**	**-0.09 (0.01)**	**0.001**	-0.76 (0.42)	0.081	0.48 (0.33)	0.141	-0.37 (0.33)	0.280	1.24 (0.72)	0.114	1.99	0.78	**5.67, 17.27**
Velocity mean return	**5.34 (0.20)**	**0.001**	**-0.01 (0.00)**	**0.001**	0.02 (0.08)	0.8	-0.03 (0.06)	0.581	-0.08 (0.06)	0.150	0.17 (0.13)	0.198	0.36	0.70	**5.94, 18.44**
Velocity max return	**6.40 (0.19)**	**0.001**	**-0.01 (0.00)**	**0.001**	0.05 (0.07)	0.482	-0.11 (0.05)	0.054	-0.07 (0.05)	0.176	0.17 (0.12)	0.169	0.35	0.76	**5.84, 18.05**
Position error peg approach	**3.54 (0.14)**	**0.001**	**0.01 (0.00)**	**0.001**	**0.19 (0.05)**	**0.001**	0.06 (0.06)	0.339	-0.08 (0.06)	0.205	0.01 (0.09)	0.932	0.19	0.42	**3.09, 12.73**
Jerk peg approach	**-13.55 (0.81)**	**0.001**	**0.07 (0.01)**	**0.001**	**1.48 (0.29)**	**0.001**	**1.91 (0.38)**	**0.001**	-0.15 (0.38)	0.694	0.15 (0.50)	0.772	0.88	0.52	**2.57, 16.38**
Log jerk peg approach	**0.23 (0.00)**	**0.001**	**0.00 (0.00)**	**0.001**	**0.00 (0.00)**	**0.001**	**0.00 (0.00)**	**0.001**	0.00 (0.00)	0.927	0.00 (0.00)	0.969	0.00	0.68	**7.77, 23.63**
SPARC peg approach	**0.33 (0.01)**	**0.001**	**0.00 (0.00)**	**0.001**	**0.02 (0.01)**	**0.001**	**0.01 (0.01)**	**0.035**	0.00 (0.01)	0.715	0.00 (0.01)	0.698	0.02	0.50	**3.59, 12.74**
Position error hole approach	**3.01 (0.21)**	**0.001**	**0.02 (0.00)**	**0.001**	**0.40 (0.08)**	**0.001**	**0.56 (0.11)**	**0.001**	0.12 (0.11)	0.315	0.09 (0.13)	0.477	0.16	0.40	**1.25, 8.41**
Jerk hole approach	**-17.57 (1.26)**	**0.001**	**0.07 (0.01)**	**0.001**	**1.83 (0.46)**	**0.001**	0.09 (0.52)	0.859	-0.37 (0.52)	0.476	-0.35 (0.80)	0.658	1.78	0.43	**1.97, 9.31**
Log jerk hole approach	**0.22 (0.00)**	**0.001**	0.00 (0.00)	0.161	0.00 (0.00)	0.629	0.00 (0.00)	0.304	0.00 (0.00)	0.341	0.00 (0.00)	0.565	0.00	0.51	**6.78, 22.32**
SPARC hole approach	**0.39 (0.02)**	**0.001**	**0.00 (0.00)**	**0.001**	**0.02 (0.01)**	**0.006**	0.01 (0.01)	0.515	0.00 (0.01)	0.991	-0.01 (0.01)	0.490	0.03	0.51	**6.57, 22.17**
Number of movement onsets	**16.86 (0.12)**	**0.001**	0.00 (0.00)	0.118	0.02 (0.04)	0.703	-0.01 (0.06)	0.869	0.01 (0.06)	0.855	0.05 (0.07)	0.532	0.12	0.06	**1.21, 8.48**
Number of movement ends	**16.43 (0.17)**	**0.001**	**0.00 (0.00)**	**0.025**	0.05 (0.06)	0.389	-0.03 (0.09)	0.721	-0.04 (0.09)	0.655	**0.41 (0.10)**	**0.001**	0.00	0.05	**3.84, 21.98**
Dropped pegs	**0.26 (0.00)**	**0.001**	**0.00 (0.00)**	**0.001**	0.00 (0.00)	1	0.00 (0.00)	0.374	**0.00 (0.00)**	**0.002**	**0.00 (0.00)**	**0.001**	0.00	0.24	**3.93, 17.99**

Supplementary Table 5: Continued.

Sensor-based metric	Fixed effects												Subject-specific effects	R^2	Model quality
	(Intercept)		Age		Sex		Tested side		Hand dominance		Stereo vision		(Intercept)		
	Estimate (SE)	p-value	Estimate (SE)	p-value	Estimate (SE)	p-value	Estimate (SE)	p-value	Estimate (SE)	p-value	Estimate (SE)	p-value	SD		C1 (%), C2 (%)
Haptic collisions mean transport	**-1.50 (0.32)**	**0.001**	**0.01 (0.00)**	**0.001**	-0.17 (0.12)	0.177	-0.14 (0.11)	0.215	0.12 (0.11)	0.307	0.23 (0.21)	0.289	0.54	0.55	**7.05, 24.00**
Haptic collisions max transport	-0.47 (0.26)	0.079	**0.01 (0.00)**	**0.001**	**-0.25 (0.10)**	**0.020**	-0.09 (0.09)	0.317	-0.01 (0.09)	0.888	0.20 (0.17)	0.198	0.42	0.52	**6.91, 21.97**
Haptic collisions mean return	**0.25 (0.00)**	**0.001**	**0.00 (0.00)**	**0.019**	0.00 (0.00)	0.070	0.00 (0.00)	0.702	**0.00 (0.00)**	**0.027**	0.00 (0.00)	0.915	0.00	0.50	**6.07, 20.78**
Haptic collisions max return	0.57 (0.41)	0.197	0.00 (0.00)	0.786	**-0.51 (0.15)**	**0.002**	**-0.34 (0.16)**	**0.039**	0.10 (0.16)	0.573	-0.11 (0.26)	0.679	0.60	0.35	7.64, 26.05
Force mean transport	**2.34 (0.20)**	**0.001**	**-0.01 (0.00)**	**0.012**	**-0.27 (0.08)**	**0.003**	0.03 (0.05)	0.558	0.09 (0.05)	0.078	-0.05 (0.14)	0.716	0.39	0.80	**4.21, 15.86**
Force max. transport	**2.59 (0.21)**	**0.001**	**-0.01 (0.00)**	**0.012**	**-0.29 (0.08)**	**0.001**	0.04 (0.05)	0.444	0.06 (0.05)	0.237	-0.06 (0.14)	0.688	0.42	0.81	**3.73, 14.13**
Force rate mean transport	**3.30 (0.36)**	**0.001**	**-0.01 (0.00)**	**0.002**	**-0.50 (0.14)**	**0.001**	0.18 (0.09)	0.053	0.06 (0.09)	0.528	-0.16 (0.24)	0.485	0.68	0.80	**2.70, 10.44**
Force rate max. transport	5.72 (0.38)	**0.001**	-0.01 (0.00)	**0.001**	-0.65 (0.15)	**0.001**	0.14 (0.09)	0.125	0.16 (0.09)	0.082	-0.24 (0.25)	0.374	0.74	**0.84**	**2.98, 10.49**
Force mean return	0.34 (0.22)	0.139	**0.01 (0.00)**	**0.001**	0.03 (0.08)	0.755	**0.25 (0.11)**	**0.035**	0.09 (0.11)	0.458	-0.03 (0.13)	0.823	0.16	0.17	**5.58, 18.73**
Force max. return	**0.26 (0.00)**	**0.001**	**0.00 (0.00)**	**0.002**	0.00 (0.00)	0.790	0.00 (0.00)	0.122	0.00 (0.00)	0.448	0.00 (0.00)	0.747	0.00	0.47	**4.14, 14.57**
Force rate mean return	**0.97 (0.20)**	**0.001**	0.00 (0.00)	0.563	-0.10 (0.08)	0.203	**0.18 (0.07)**	**0.017**	0.08 (0.07)	0.225	0.01 (0.13)	0.955	0.35	0.53	**6.48, 22.78**
Force rate max. return	1.55 (0.11)	**0.001**	0.00 (0.00)	**0.001**	-0.11 (0.04)	**0.008**	0.14 (0.04)	**0.002**	0.02 (0.04)	0.546	-0.04 (0.07)	0.523	0.18	**0.61**	**4.98, 18.57**
Force mean peg approach	**1.96 (0.21)**	**0.001**	**-0.01 (0.00)**	**0.026**	**-0.27 (0.08)**	**0.002**	-0.10 (0.07)	0.110	0.08 (0.07)	0.245	-0.16 (0.14)	0.228	0.37	0.65	**3.54, 12.43**
Force max. peg approach	**2.64 (0.22)**	**0.001**	0.00 (0.00)	0.130	**-0.24 (0.08)**	**0.006**	0.01 (0.05)	0.791	0.04 (0.05)	0.469	-0.12 (0.15)	0.444	0.42	0.80	**3.84, 14.20**
Force rate mean peg approach	**3.68 (0.28)**	**0.001**	**-0.02 (0.00)**	**0.001**	**-0.52 (0.11)**	**0.001**	-0.08 (0.07)	0.267	0.07 (0.07)	0.312	-0.27 (0.19)	0.159	0.54	0.83	**3.13, 11.09**
Force rate max. peg approach	**5.17 (0.31)**	**0.001**	**-0.01 (0.00)**	**0.009**	**-0.44 (0.12)**	**0.001**	0.00 (0.08)	0.955	0.07 (0.08)	0.403	-0.13 (0.21)	0.540	0.61	0.82	**3.13, 10.87**
Force mean hole approach	**1.82 (0.15)**	**0.001**	0.00 (0.00)	0.064	**-0.17 (0.06)**	**0.006**	-0.01 (0.04)	0.843	0.06 (0.04)	0.160	-0.07 (0.10)	0.467	0.28	0.76	**4.84, 18.59**
Force max. hole approach	**1.99 (0.16)**	**0.001**	0.00 (0.00)	0.204	**-0.17 (0.06)**	**0.012**	0.01 (0.04)	0.870	0.08 (0.04)	0.067	-0.10 (0.11)	0.361	0.30	0.75	**5.33, 20.21**
Force rate mean hole approach	**30.63 (4.59)**	**0.001**	**-0.21 (0.05)**	**0.001**	**-6.80 (1.78)**	**0.001**	0.92 (1.15)	0.427	**3.91 (1.15)**	**0.002**	-3.56 (3.06)	0.253	8.89	0.83	**4.57, 15.92**
Force rate max. hole approach	**5.25 (0.36)**	**0.001**	**-0.01 (0.00)**	**0.011**	**-0.48 (0.14)**	**0.001**	0.01 (0.10)	0.951	**0.26 (0.10)**	**0.010**	-0.16 (0.24)	0.524	0.67	0.73	**5.81, 21.28**
Force rate number of peaks transport	**1.17 (0.19)**	**0.001**	**0.02 (0.00)**	**0.001**	0.12 (0.07)	0.069	-0.07 (0.08)	0.421	0.09 (0.08)	0.259	-0.14 (0.12)	0.256	0.25	0.47	**7.11, 23.64**
Force rate spectral arc length transport	**0.42 (0.02)**	**0.001**	**0.00 (0.00)**	**0.001**	0.00 (0.01)	0.627	-0.02 (0.01)	0.075	-0.01 (0.01)	0.297	-0.01 (0.02)	0.606	0.04	0.42	**6.97, 23.19**
Force rate number of peaks return	**1.86 (0.12)**	**0.001**	**0.00 (0.00)**	**0.002**	**-0.15 (0.04)**	**0.002**	-0.06 (0.04)	0.173	0.02 (0.04)	0.722	-0.12 (0.07)	0.116	0.18	0.48	**7.53, 23.94**
Force rate spectral arc length return	**0.63 (0.04)**	**0.001**	**0.00 (0.00)**	**0.001**	-0.03 (0.02)	0.075	0.02 (0.02)	0.176	0.02 (0.02)	0.155	-0.02 (0.03)	0.513	0.06	0.43	**8.11, 24.87**
Force rate number of peaks peg approach	**1.24 (0.01)**	**0.001**	**0.00 (0.00)**	**0.001**	**0.02 (0.00)**	**0.001**	**0.01 (0.00)**	**0.007**	0.00 (0.00)	0.941	0.01 (0.01)	0.275	0.01	0.66	**3.30, 14.23**
Force rate spectral arc length peg approach	**0.80 (0.01)**	**0.001**	**0.00 (0.00)**	**0.001**	**0.02 (0.00)**	**0.001**	0.01 (0.00)	0.237	0.00 (0.00)	0.563	0.01 (0.01)	0.182	0.01	0.48	**3.86, 15.46**
Force rate number of peaks hole approach	**0.82 (0.10)**	**0.001**	**0.01 (0.00)**	**0.001**	**0.13 (0.04)**	**0.006**	0.02 (0.04)	0.702	-0.01 (0.04)	0.851	0.02 (0.06)	0.772	0.16	0.57	**2.17, 9.08**
Force rate spectral arc length hole approach	**0.26 (0.03)**	**0.001**	**0.00 (0.00)**	**0.001**	**0.04 (0.01)**	**0.001**	0.00 (0.01)	0.781	-0.01 (0.01)	0.376	-0.01 (0.02)	0.594	0.05	0.62	**2.28, 11.56**
Force rate number of peaks buildup	0.00 (0.02)	0.987	**0.00 (0.00)**	**0.008**	0.00 (0.01)	0.494	0.02 (0.01)	0.062	**0.02 (0.01)**	**0.032**	**-0.03 (0.01)**	**0.012**	0.02	0.12	4.58, 35.23
Force rate spectral arc length buildup	**0.04 (0.00)**	**0.001**	**0.00 (0.00)**	**0.001**	**0.00 (0.00)**	**0.006**	0.00 (0.00)	0.733	0.00 (0.00)	0.262	0.00 (0.00)	0.177	0.00	0.40	6.64, 25.87
Force buildup duration	**-2.66 (0.19)**	**0.001**	**0.01 (0.00)**	**0.002**	**0.26 (0.07)**	**0.001**	-0.01 (0.06)	0.878	0.01 (0.06)	0.904	-0.16 (0.12)	0.223	0.33	0.61	**6.16, 19.68**
Force rate number of peaks release	0.00 (0.01)	0.795	0.00 (0.00)	0.226	0.00 (0.00)	0.819	0.01 (0.00)	0.207	0.00 (0.00)	0.748	0.00 (0.01)	0.687	0.00	0.02	6.87, 71.30
Force rate spectral arc length release	**1.04 (0.04)**	**0.001**	**0.00 (0.00)**	**0.001**	**0.05 (0.01)**	**0.001**	-0.02 (0.01)	0.214	0.00 (0.01)	0.882	0.02 (0.02)	0.325	0.05	0.56	**3.53, 12.34**
Force release duration	**-2.32 (0.18)**	**0.001**	0.00 (0.00)	0.170	**0.17 (0.07)**	**0.012**	0.12 (0.07)	0.124	-0.04 (0.07)	0.617	-0.13 (0.11)	0.279	0.25	0.31	**8.05, 24.38**
Task completion time	**1.13 (0.01)**	**0.001**	**0.00 (0.00)**	**0.001**	**0.02 (0.00)**	**0.001**	**0.01 (0.00)**	**0.033**	0.00 (0.00)	0.665	0.00 (0.01)	0.633	0.01	0.73	**4.13, 17.21**
Simulated Gaussian noise	**4.42 (0.12)**	**0.001**	0.00 (0.00)	0.851	0.04 (0.04)	0.302	-0.10 (0.06)	0.118	-0.02 (0.06)	0.784	0.03 (0.07)	0.651	0.10	0.03	12.00, 35.29

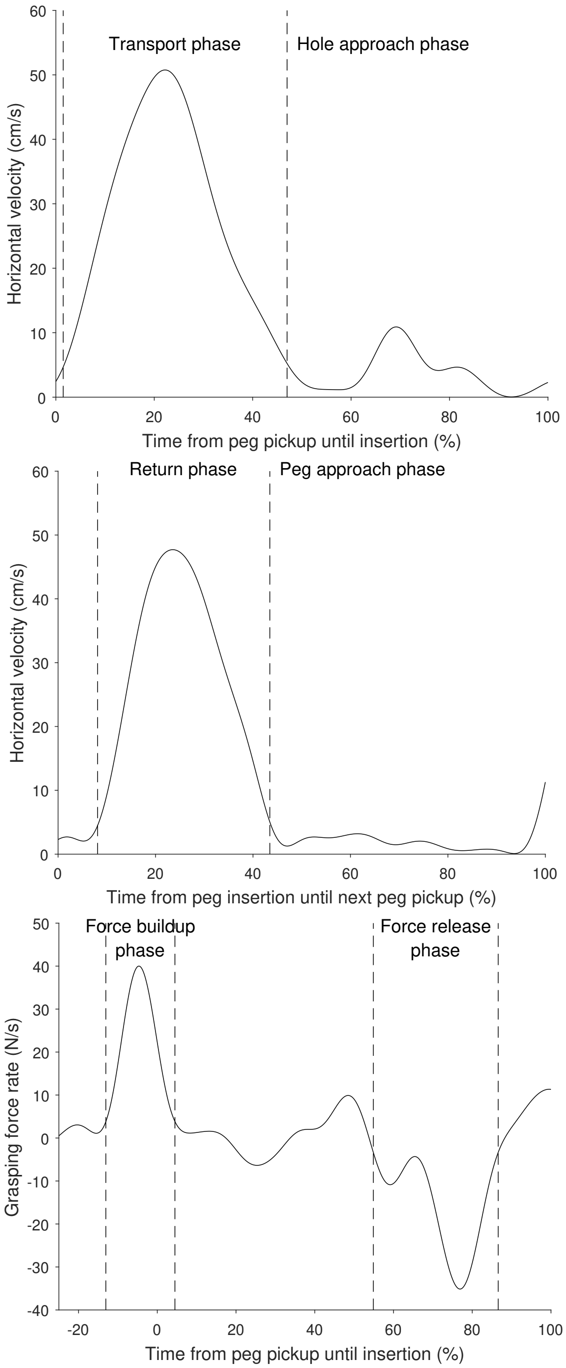

Supplementary Figure 1: **Temporal segmentation of kinematic and kinetic trajectories.** Representative example from one neurologically intact subject (49yrs, female, tested hand left, dominant hand right).

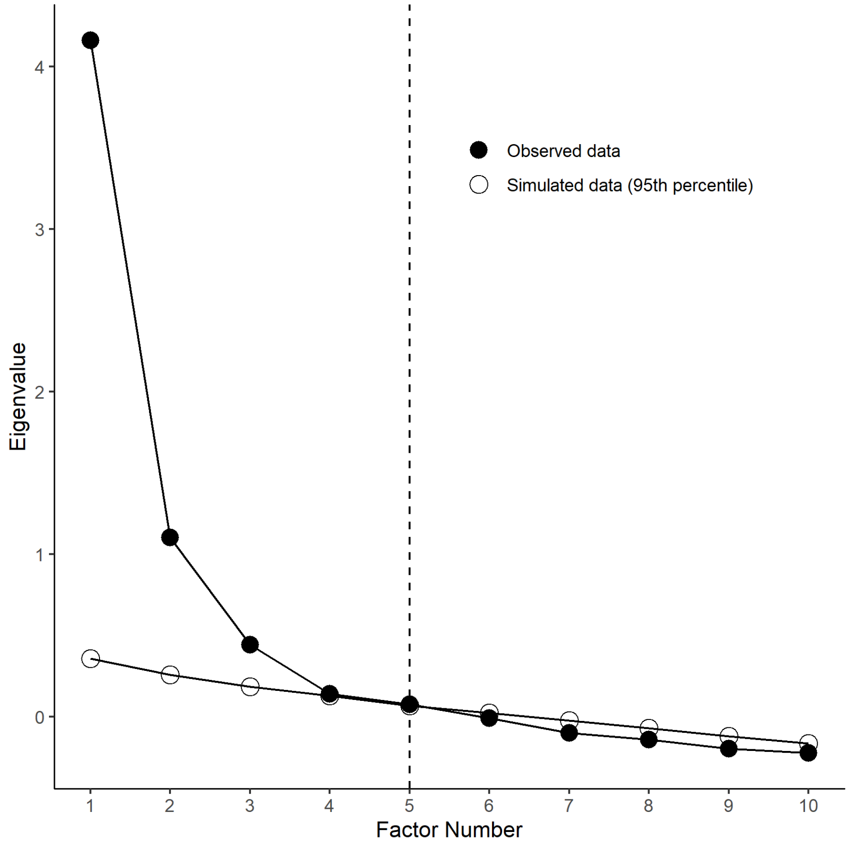

Supplementary Figure 3: Scree plot for estimating the number of latent variables k in the factor analysis. Parallel analysis was used to simulate a lower bound of an eigenvalues magnitude that each eigenvalue in the observed data needs to fulfill. The chosen number of factors was set to five accordingly.

References

[1] Robert Tibshirani. Regression Shrinkage and Selection via the Lasso. *Journal of the Royal Statistical Society*, 58(1):267–288, 1996.

[2] Leo Breiman. Random Forests. *Machine Learning*, 45(1):5–32, aug 2001.

[3] Berk Ustun and Cynthia Rudin. Supersparse linear integer models for optimized medical scoring systems. *Machine Learning*, 102(3):349–391, 2016.

[4] Isabelle Guyon and André Elisseeff. An Introduction to Variable and Feature Selection. *Journal of Machine Learning Research (JMLR)*, 3(3):1157–1182, 2003.
